# Cross-Species Behavioral Representation Learning Using Domain-Adversarial Adaptation on Wearable IMU Signals

**DOI:** 10.3390/biomimetics11070496

**Published:** 2026-07-15

**Authors:** Çiğdem İnan Acı, Furkan Say, Esin Ayşe Zaimoğlu

**Affiliations:** 1 Department of Computer Engineering, Mersin University, 33343 Mersin, Turkey; 2 Department of Information Systems Engineering, Sakarya University, 54050 Sakarya, Turkey; esinzaimoglu@sakarya.edu.tr

**Keywords:** activity recognition, domain adaptation, wearable sensors, biomimetic representation learning, cross-species transfer, deep learning, imbalanced learning

## Abstract

Animal locomotion exhibits highly structured temporal dynamics despite significant inter-species biomechanical and anatomical discrepancies. While wearable inertial measurement unit (IMU) sensors and deep learning have advanced animal activity recognition, existing systems remain largely species-dependent, requiring large-scale labeled datasets for each deployment. To address this, we propose a biomimetic cross-species behavioral representation learning framework that extracts transferable locomotor structures from heterogeneous IMU signals. The proposed methodology integrates behavioral ontology harmonization, imbalance-aware augmentation, and semi-supervised domain-adversarial adaptation to reduce inter-species distributional discrepancies. Unlike conventional classification models, our dual-head architecture enables simultaneous processing of multi-label and single-label behavioral structures across anatomically diverse species. Extensive experiments conducted on dog, goat, and horse datasets demonstrate that the proposed framework significantly improves cross-species transferability, achieving a mean Macro-F1 score of 0.711 compared to 0.345 for direct transfer learning. Furthermore, we show that sparse target supervision is critical for stabilizing adversarial adaptation. K-Means head adaptation partially mitigated fully unsupervised negative transfer. However, its performance remained below semi-supervised Domain-Adversarial Neural Network (DANN), indicating that sparse target supervision is still necessary for stable cross-species adaptation. These findings suggest that biologically motivated locomotor similarity can support cross-species behavioral transfer, although external validation on additional species, sensor placements, and deployment conditions is still required.

## 1. Introduction

Animal locomotion exhibits highly structured temporal dynamics despite substantial anatomical and biomechanical differences among species. Behaviors such as walking, standing, grazing, trotting, resting, and feeding emerge from coordinated locomotor patterns shaped by neuromuscular and biomechanical adaptation processes. In recent years, wearable inertial measurement unit (IMU) sensors have enabled continuous monitoring of these behavioral patterns under natural conditions, supporting the development of intelligent animal activity recognition systems for livestock management, animal welfare assessment, veterinary monitoring, and precision farming applications [1,2,3]. Advances in deep learning have further accelerated the ability to model temporal behavioral signals directly from raw multivariate accelerometer streams without extensive handcrafted feature engineering [4,5,6]. Nevertheless, most existing wearable sensing systems remain strongly species-dependent and generally require large-scale labeled datasets collected under fixed sensor configurations and controlled environmental conditions.

Early animal activity recognition studies primarily relied on handcrafted statistical and frequency-domain features extracted from accelerometer signals combined with conventional machine learning algorithms such as Random Forests, Support Vector Machines, and k-Nearest Neighbors for species-specific activity classification [7,8,9]. These approaches demonstrated that wearable sensors could successfully distinguish fundamental locomotor behaviors in sheep, cattle, goats, horses, and dogs under controlled experimental settings [7,10,11,12]. Subsequently, deep learning architectures, particularly Convolutional Neural Networks (CNNs), Long Short-Term Memory (LSTM) networks, Bidirectional LSTMs, and Gated Recurrent Units (GRUs), were introduced to automatically learn discriminative temporal motion representations directly from raw sensor streams [5,13,14,15]. Attention-based recurrent models and transformer-inspired architectures further improved recognition performance by capturing both local motion dynamics and long-term temporal dependencies within animal movement patterns [16,17]. Despite these advances, most existing approaches still assume that training and test data originate from the same species and sensing distribution, limiting their generalization capability under heterogeneous real-world conditions.

Recent studies have demonstrated that animal behavior recognition performance is affected not only by model architecture but also by sensor placement, sampling frequency, segmentation strategy, behavioral imbalance, and inter-individual variability [8,18,19]. In practical deployment scenarios, anatomical differences, varying sensor attachment positions, and heterogeneous environmental conditions introduce substantial distributional discrepancies between datasets collected from different animal species [20]. Consequently, each newly introduced species or sensing condition often requires expensive manual annotation and model retraining. Furthermore, many animal behaviors exhibit hierarchical and partially overlapping structures, making conventional single-label classification frameworks insufficient for representing complex behavioral dynamics [21]. These limitations indicate that current wearable sensing systems remain highly dependent on domain-specific supervision and often fail to generalize under severe anatomical, behavioral, and environmental heterogeneity.

To overcome these limitations, recent research has increasingly focused on transfer learning, domain adaptation, and semi-supervised learning strategies capable of extracting transferable behavioral representations from heterogeneous sensor data [22,23,24,25]. Among these approaches, Domain-Adversarial Neural Networks (DANNs) have attracted considerable attention because of their ability to reduce domain discrepancies through adversarial feature alignment [22,23]. Conditional adversarial adaptation and pseudo-labeling strategies have further improved adaptation performance under limited target supervision [24,25]. In parallel, several studies explored federated learning frameworks for scalable and privacy-preserving livestock monitoring across distributed sensing infrastructures [26]. Data augmentation and imbalance-aware learning methods, including Generative Adversarial Network (GAN)-based augmentation, Multi-Label Synthetic Minority Over-sampling Technique (MLSMOTE), and synthetic oversampling approaches, have also been proposed to address severe class imbalance problems frequently observed in wearable animal behavior datasets [27,28,29,30]. In addition, compact time-series representation techniques such as catch22 descriptors have been introduced to improve computational efficiency while preserving discriminative locomotor characteristics [31]. However, most existing studies continue to formulate animal behavior analysis primarily as a closed-set classification problem rather than a biomimetic representation learning problem centered on discovering transferable locomotor structures shared across species. Therefore, the present study focuses specifically on wearable IMU-based animal activity recognition and cross-species domain adaptation, rather than on broader AI applications outside the animal-sensing context.

Cross-species behavioral intelligence remains particularly underexplored in wearable sensing research. Existing domain adaptation studies mainly investigate transfer across sensor positions or environmental conditions within a single species rather than between anatomically heterogeneous animals [23,32]. Moreover, behavioral annotation schemes frequently differ between datasets, leading to semantic inconsistencies that complicate direct representation transfer across species [32]. Recent cross-species knowledge-sharing frameworks demonstrated that biologically shared locomotor structures can partially improve universal activity recognition performance across multiple animal species [33]. Nevertheless, severe inter-species biomechanical discrepancies, sparse target annotations, and behavioral imbalance frequently cause negative transfer and unstable adaptation behavior under realistic deployment conditions [25]. However, most of these methods evaluate adaptation at the level of domain alignment or target-domain accuracy, without explicitly examining whether the learned representations preserve behavior-level transferability under heterogeneous annotation structures. This limitation is particularly important when one dataset follows a multi-label behavioral scheme while another follows a mutually exclusive single-label scheme. Therefore, developing biologically meaningful and transferable latent representations capable of preserving shared locomotor dynamics while adapting to species-specific behavioral patterns remains a major open challenge in biomimetic wearable sensing systems.

In this study, we propose a biomimetic cross-species behavioral representation learning framework for transferable animal activity recognition using wearable IMU signals collected from heterogeneous animal species. The proposed framework integrates semi-supervised domain adaptation, adversarial feature alignment, imbalance-aware learning, and temporal deep representation learning to reduce inter-species distribution discrepancies while preserving biologically shared locomotor structures. Unlike conventional activity classification systems that primarily optimize within-domain prediction accuracy, the proposed approach focuses on learning species-invariant latent behavioral representations that remain transferable across heterogeneous anatomical and environmental conditions. The framework incorporates a dual-head architecture capable of jointly processing multi-label and single-label behavioral structures across different species datasets.

In addition, Behavioral Transferability Similarity Score (BTSS)-based label harmonization, Principal Component Analysis (PCA)-based representation optimization, and imbalance-aware augmentation strategies are integrated into the learning pipeline to improve cross-species adaptation robustness. Extensive experiments are conducted under transfer learning and semi-supervised domain adaptation settings with varying target-label availability using multiple wearable IMU datasets containing heterogeneous behavioral distributions and annotation structures. The study further investigates negative transfer behavior under fully unsupervised conditions and analyzes the effects of sparse supervision on adversarial adaptation performance. Experimental results demonstrate that biologically informed adversarial representation learning substantially improves cross-species transferability while reducing dependence on large-scale labeled target datasets, highlighting the potential of biomimetic representation learning for next-generation adaptive animal behavior intelligence systems.

In this study, the term *biomimetic* does not refer to the explicit computational emulation of animal neural control mechanisms. Rather, it refers to a biologically motivated representation-learning strategy in which shared locomotor regularities across species are used as the basis for cross-species behavioral transfer. Therefore, the biomimetic interpretation is limited to locomotor-structure-inspired abstraction and should not be interpreted as evidence of direct neurobiological modeling.

The main contributions of this study are as follows:

1. A dual-head cross-species learning architecture is proposed to jointly handle multi-label dog annotations and single-label goat/horse annotations within a unified transfer-learning framework.
2. A BTSS- and Jensen–Shannon Divergence (JSD)-guided behavioral ontology harmonization strategy is used to map heterogeneous species-specific labels into six transferable behavior classes.
3. A semi-supervised DANN framework is evaluated across six bidirectional source–target transfer settings, four temporal architectures, and three training stages, resulting in 72 model-stage evaluations.
4. The role of sparse target supervision is explicitly analyzed through target-supervision weight ablation, showing that even minimal target-label information can prevent severe negative transfer.
5. The contribution of preprocessing components is quantified through an ablation analysis covering class balancing, PCA transformation, noise cleaning, and minority-window boosting.

## 2. Materials and Methods

This study proposes a biomimetic cross-species behavioral representation learning framework for transferable animal activity recognition using wearable inertial measurement unit (IMU) sensor data collected from heterogeneous animal species. The proposed methodology integrates behavioral ontology harmonization, imbalance-aware preprocessing, adversarial domain adaptation, and temporal deep representation learning within a unified semi-supervised learning pipeline. The overall framework was designed to investigate whether biologically shared locomotor structures can be preserved across anatomically distinct species while reducing dependence on large-scale labeled target datasets.

### 2.1. Datasets and Behavioral Ontology Harmonization

All datasets used in this study were obtained from publicly available wearable IMU datasets reported in previous studies. No new animal experiment was conducted. The dog dataset follows a multi-label annotation scheme, whereas the goat and horse datasets follow single-label multi-class annotation schemes. Therefore, the experimental design was constructed not only as a cross-species transfer problem but also as a heterogeneous-label-structure adaptation problem.

Three publicly available wearable IMU datasets collected from dogs, goats, and horses were used in this study [11,12,34]. These datasets were selected because they contain heterogeneous locomotor behaviors, distinct anatomical movement patterns, and different labeling structures, thereby providing a suitable benchmark for evaluating cross-species behavioral transferability under realistic domain discrepancy conditions. The dog dataset consisted of multi-label behavioral annotations collected using collar-mounted wearable sensors [11], whereas the goat and horse datasets contained single-label locomotor behavior annotations acquired from body-mounted IMU devices [12,18]. Consequently, substantial inter-species differences existed not only in movement biomechanics but also in annotation semantics, sensor placement, sampling configuration, and behavioral distribution characteristics. Detailed characteristics of the datasets used in this study are summarized in Table 1.

Because the datasets were collected in different original studies, they differ in subject composition, annotation protocol, and behavioral distribution. These differences were not removed from the evaluation design; instead, they were treated as part of the cross-species domain shift to be addressed by the proposed adaptation framework.

To establish a common behavioral representation space across species, a BTSS-based ontology harmonization procedure was applied prior to model training [33,35]. Initially, all species-specific behavior labels were analyzed using Jensen–Shannon Divergence (JSD)-based behavioral similarity estimation to quantify inter-class transferability relationships between datasets [35,36]. Behaviors exhibiting semantically and biomechanically similar locomotor characteristics were subsequently grouped into unified transferable behavioral categories [33]. Through this procedure, species-specific annotations were reduced into six common behavioral classes shared across datasets. This harmonization stage enabled direct cross-species comparison and domain adaptation by minimizing semantic inconsistencies between heterogeneous annotation protocols [32].

The harmonization procedure consisted of four steps. First, all original behavior labels were extracted from the dog, goat, and horse datasets. Second, window-level feature distributions were generated for each original label. Third, pairwise behavioral similarity was estimated using JSD, and semantically compatible labels with similar locomotor characteristics were grouped. Fourth, labels observed in only one species, labels representing composite or ambiguous behavioral states, and labels with insufficient transferable samples were excluded from the unified ontology. The raw-label-level JSD matrices for the dog–goat, dog–horse, and goat–horse comparisons are provided in Supplementary Figures S1–S3, while the unified-class-level validation matrices are provided in Supplementary Figures S4–S6.

The resulting behavioral ontology included locomotor and stationary behavior categories that were consistently observable across all species [21,33]. Since the dog dataset contained simultaneous multi-label behavioral annotations while the goat and horse datasets contained mutually exclusive single-label annotations [11,12,18], the proposed framework adopted a hybrid dual-head learning strategy capable of jointly processing both multi-label and single-label classification structures within a unified representation learning pipeline. This design enabled transferable behavioral modeling without discarding species-specific annotation characteristics. The final unified behavioral ontology and cross-species label mappings are presented in Table 2. The JSD-based raw-label similarity matrices and unified-class ontology validation matrices are provided in Supplementary Figures S1–S6.

For the dog dataset, the original behavior annotation columns were merged into a six-dimensional multi-label target vector after ontology harmonization. For the goat and horse datasets, each window was assigned to one mutually exclusive class within the same six-class ontology.

The unified ontology was designed for transfer-level behavioral abstraction rather than fine-grained ethological classification. Therefore, some original labels were intentionally grouped into broader locomotor categories to create a shared cross-species label space. For example, jumping, playing, galloping, and running were grouped under high-intensity locomotion, whereas sniffing was grouped with low-intensity locomotion when its window-level motion pattern was closer to walking-like movement. Although JSD and BTSS were used to support these mappings quantitatively, they do not fully replace biological validation. Thus, ontology harmonization may improve transferability while reducing behavioral specificity.

### 2.2. Behavioral Transferability Similarity Score

To make the ontology harmonization and class-wise adaptation procedure explicit, the Behavioral Transferability Similarity Score (BTSS) was formally defined as a JSD-derived class-level transferability measure. For each unified behavior class c, source-domain and target-domain feature distributions were estimated separately for each feature dimension d. Let $P_{s}(c,d)$ and $P_{t}\left(c,d\right)$ denote the normalized histogram-based probability distributions of the source and target samples belonging to behavior class c in feature dimension d, respectively. The Jensen–Shannon divergence (JSD) between these distributions is given in Equation (1).

(1) $$JSD\left(P∥Q\right)=\frac{1}{2}D_{KL}\left(P∥M\right)+\frac{1}{2}D_{KL}\left(Q∥M\right),M=\frac{1}{2}(P+Q)$$

In Equation (1), $D_{KL}\;$denotes the Kullback–Leibler (KL) divergence. For discrete histogram bins, it is defined as given in Equation (2):

(2) $$D_{KL}\left(P∥M\right)=\sum_{i=1}^{n}P_{i}{log}_{2}(\frac{P_{i}}{M_{i}})$$

Here, i denotes the histogram-bin index, $P_{i}\;$is the probability mass of P in bin i, and $M_{i}$ is the corresponding probability mass of the mixture distribution M. Terms with $P_{i}=0$ were treated as zero in the summation. With logarithms computed in base 2, JSD is bounded in the interval [0, 1]. Unlike KL divergence, JSD is symmetric and finite because both P and Q are compared with their common mixture distribution M.

The class-level BTSS is given in Equation (3), where the JSD values are averaged across all D feature dimensions and converted into a similarity score:

(3) $$BTSS\;\left(c\right)=1-\frac{1}{D}\sum_{d=1}^{D}{JSD(P}_{s}\left(c,d\right),P_{t}(c,d))$$

Accordingly, BTSS(c)∈[0, 1], where values close to 1 indicate high cross-species similarity and stronger behavioral transferability, while values close to 0 indicate large source–target distributional discrepancy and lower transferability. Therefore, JSD was used as the underlying distributional distance measure, whereas BTSS represents a behavior-level normalized transferability score derived from JSD. In other words, JSD quantifies feature-wise distributional distance, while BTSS aggregates these distances into an interpretable class-wise transferability score.

BTSS was operationalized in two ways. First, it was used together with the semantic and biomechanical definitions of the behaviors to support the construction of the six-class shared behavioral ontology. Raw labels with low JSD and high BTSS, and with compatible operational definitions, were grouped into the same transferable behavior class. Labels with weak transferability, ambiguous behavioral meaning, or insufficient transferable samples were excluded from the unified ontology. Second, BTSS was used to weight the target-domain focal loss during DANN training. For each behavior class c, the BTSS-based loss weight is given in Equation (4):

(4) $$w\left(c\right)=\alpha \left[1-BTSS\left(c\right)\right]+(1-\alpha )$$

Here, α = 0.7 controls the contribution of the transferability term. The resulting weights were normalized across classes and multiplied by the target-domain focal loss at the sample level. In this formulation, behaviors with lower transferability receive relatively higher adaptation weight, whereas behaviors that are already highly transferable receive lower additional weighting.

### 2.3. Data Preprocessing and Imbalance-Aware Augmentation

The collected IMU datasets exhibited substantial inter-species variability in sampling characteristics, behavioral distributions, sensor placement configurations, and annotation density. In addition, severe class imbalance was observed across several behavioral categories, particularly in rare locomotor and transition behaviors. To improve representation consistency and adaptation stability, a multi-stage preprocessing pipeline was applied prior to model training. The class distributions obtained after ontology harmonization and window-level segmentation are summarized in Table 3.

Temporal segmentation was performed using a sliding window of 128 samples. At 100 Hz, this corresponds to approximately 1.28 s per window. Label homogeneity filtering was applied only during training-window construction to reduce ambiguous windows. The test partitions were kept unchanged to preserve a realistic evaluation protocol. For multi-label dog windows, label assignment was performed after merging the original behavior annotation columns into the unified six-class vector. Windows with ambiguous or inconsistent label composition were excluded only from the training set by the homogeneity filter.

To prevent subject-level data leakage, the train, validation, and test partitions were created at the animal-subject level before window generation and before any cleaning, balancing, augmentation, or PCA fitting operation. Accordingly, all windows originating from the same animal were assigned to only one partition and were never shared across training, validation, and test sets. This subject-wise partitioning strategy ensured that the model was evaluated on unseen animals rather than on temporally adjacent windows from animals already observed during training.

After subject-wise partitioning, preprocessing operations were restricted to the training partition. Noise cleaning, class balancing, synthetic sample generation, feature standardization, and PCA fitting were performed only on the training data. The validation and test partitions were transformed using the parameters learned from the training data and were not cleaned, balanced, or augmented. The subject-wise split was performed using a training/validation/test ratio of 70/15/15. Due to the small number of goat subjects, the 70/15/15 split ratio was implemented using the nearest feasible subject-level allocation, while keeping all windows from the same animal within a single partition.

The exact subject-level allocation used in the train/validation/test split is reported in Table 4. For the dog dataset, 45 subjects were divided into 31 training, 7 validation, and 7 test subjects. For the goat dataset, the small number of available animals required the nearest feasible subject-level allocation, resulting in 3 training, 1 validation, and 1 test subject. For the horse dataset, 18 subjects were divided into 12 training, 3 validation, and 3 test subjects. All windows from the same animal were assigned to only one partition. Therefore, no animal-level leakage occurred across the training, validation, and test partitions. However, because the goat dataset contains only five animals, goat-target results should be interpreted with caution and are reported as a limited-sample transfer scenario rather than as a statistically broad species-level validation.

Initially, noisy and potentially mislabeled samples were removed using Edited Nearest Neighbors (ENN) and Tomek Links filtering methods [37,38]. ENN eliminates samples whose class labels disagree with the majority labels of their nearest neighbors, thereby reducing local class overlap and noisy decision boundaries [37]. Tomek Links were subsequently employed to identify ambiguous inter-class boundary samples and improve class separability within the feature space [38]. This cleaning stage was particularly important for reducing inter-species distributional noise before adversarial adaptation. The retained and removed training windows after the cleaning stage are reported in Table 5.

Following noise reduction, imbalance-aware augmentation strategies were applied to address the highly skewed behavioral distributions observed across datasets. Since the dog dataset contained multi-label annotations, the Multi-Label Synthetic Minority Over-sampling Technique (MLSMOTE) was employed to generate synthetic minority samples while preserving label co-occurrence structures [29]. MLSMOTE extends conventional SMOTE-based augmentation to multi-label learning problems by synthesizing feature vectors and associated label sets simultaneously [29]. For single-label datasets, additional synthetic augmentation was performed using Conditional Wasserstein Generative Adversarial Networks with Gradient Penalty (cWGAN-GP) [28,39]. The adversarial augmentation framework was designed to generate realistic synthetic IMU sequences while improving minority-class representation diversity and reducing mode collapse during GAN training [28].

For the single-label goat and horse datasets, cWGAN-GP augmentation was applied only to the training partition and only for minority classes below the class-balancing target. The conditioning variable was the harmonized six-class behavior label, encoded as a one-hot class vector. The generator received a Gaussian noise vector (dimension 32) concatenated with this class vector and produced synthetic samples in the PCA-transformed feature space. The critic received either a real or generated PCA feature vector concatenated with the same one-hot class condition and returned a scalar Wasserstein score. Both the generator and critic were implemented as fully connected multilayer perceptrons; the generator used a (32 + 6) → 128 → 128 → d architecture with LeakyReLU(0.2) activations, and the critic used a (d + 6) → 128 → 128 → 1 architecture with LeakyReLU(0.2) activations and dropout (*p* = 0.2), where d is the PCA feature dimensionality (components retained at 99% explained variance) and 6 is the number of harmonized behavior classes. The cWGAN-GP models were trained for 800 generator update steps (mini-batch size 256) with the Adam optimizer (learning rate 1 × 10^−4^, β_1_ = 0.0, β_2_ = 0.9), using a gradient-penalty coefficient of 10 and 5 critic updates per generator update. As is standard for WGAN-GP, training length is specified as the number of generator update iterations rather than epochs, since each iteration draws a randomly sampled mini-batch. Synthetic samples were accepted only until the predefined geometric-mean class target was reached, with each augmented (minority) class receiving at most 20% synthetic samples relative to its real count, while majority classes were undersampled to the same target rather than augmented. Synthetic sample quality was checked by applying the same post-generation ENN-based cleaning procedure used in the preprocessing pipeline. The validation and test partitions were never augmented, cleaned, or used during cWGAN-GP fitting; therefore, all reported test results were obtained on unchanged real target-domain samples.

After augmentation, Principal Component Analysis (PCA) was applied for dimensionality reduction and representation stabilization prior to deep feature extraction [40]. PCA was used to reduce redundancy within high-dimensional temporal IMU feature representations while preserving dominant locomotor variance across species. Four different PCA fitting strategies were evaluated, including Dog-PCA, Goat-PCA, Horse-PCA, and combined PCA configurations, in order to analyze the effect of source-domain representation structure on transfer performance. This stage additionally aimed to reduce species-specific feature dominance and improve adversarial alignment stability during domain adaptation training. The effect of PCA strategy was therefore evaluated experimentally rather than assumed a priori, and the final representation setting was selected according to transfer performance and representation stability.

To prevent evaluation leakage, all cleaning, class balancing, synthetic sample generation, and PCA fitting operations were applied only to the training partition. The test set was not cleaned, balanced, augmented, or used for PCA fitting. Therefore, the reported test performance reflects adaptation to the original target-domain distribution rather than performance on a synthetically balanced or cleaned test set.

### 2.4. Cross-Species Behavioral Representation Learning Framework

**Figure 1:**
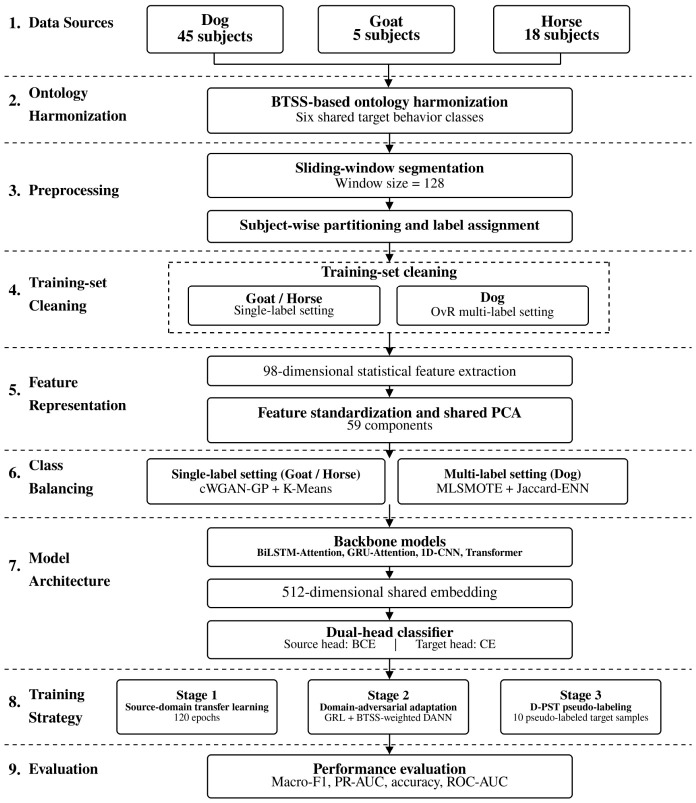
Overall architecture of the proposed cross-species behavioral representation learning framework. The pipeline begins with public dog, goat, and horse IMU datasets, followed by BTSS-based ontology harmonization, subject-wise partitioning, training-set-only preprocessing, dual-head temporal representation learning, domain-adversarial adaptation, and evaluation on unchanged target test partitions.

The proposed framework is a semi-supervised cross-species behavioral representation learning pipeline that integrates temporal feature extraction, adversarial domain adaptation, dual-head behavioral classification, and sparse-label target adaptation. The overall architecture of the framework is shown in Figure 1.

To investigate the effect of temporal representation learning capacity on cross-species transferability, four different deep learning architectures were evaluated as feature extractors: Bidirectional Long Short-Term Memory with attention (BiLSTM-Attention), Gated Recurrent Unit with attention (GRU-Attention), Transformer, and one-dimensional Convolutional Neural Network (CNN1D) architectures [13,14,41,42]. Recurrent architectures were selected because of their ability to model long-term temporal dependencies within sequential locomotor signals [13,14]. Attention mechanisms were incorporated to improve temporal saliency estimation and emphasize behaviorally informative motion segments during representation learning [16]. Transformer-based temporal modeling was additionally evaluated due to its self-attention-based global sequence learning capability, whereas CNN1D architectures were included as computationally efficient local temporal feature extractors [43]. The common architectural settings and training hyperparameters used for all evaluated models are summarized in Table 6. The evaluated training stages were transfer learning (TL), DANN adaptation, and Dynamic Pseudo Self-Training (D-PST). Binary cross-entropy (BCE) and categorical cross-entropy (CE) denote the loss families used for multi-label and single-label heads, respectively.

To reduce inter-species distribution discrepancies during transfer learning, DANN-based adaptation was employed [22]. The DANN framework integrates a Gradient Reversal Layer (GRL) between the feature extractor and the domain discriminator to encourage the extraction of species-invariant latent representations. During training, the feature extractor minimizes behavioral classification loss while simultaneously maximizing domain confusion through adversarial optimization. This adversarial process enables latent features originating from different animal species to become less distinguishable within the shared representation space, thereby improving transferability across heterogeneous domains.

**Figure 2:**
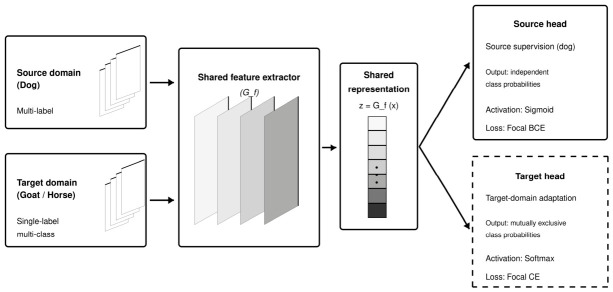
Hybrid dual-head classification mechanism for heterogeneous annotation structures.

Since the dog dataset contained multi-label annotations while the goat and horse datasets contained mutually exclusive single-label structures, the proposed framework incorporated a hybrid dual-head classification architecture. The source-domain branch employed focal binary cross-entropy loss for multi-label learning, whereas the target-domain branch used focal categorical cross-entropy loss for single-label classification [29,44]. This design enabled simultaneous optimization across heterogeneous annotation structures without discarding species-specific behavioral information. Focal loss functions were selected to reduce the influence of dominant classes and improve learning stability under severe behavioral imbalance conditions [29]. The hybrid dual-head classification mechanism used for jointly processing multi-label and single-label behavioral structures is illustrated in Figure 2.

The dual-head design was required because the source and target domains did not always share the same annotation structure. In Dog → Goat and Dog → Horse transfer settings, the multi-label dog predictions were not directly converted into a single-label target prediction. Instead, the dog source head used independent sigmoid outputs and focal binary cross-entropy to supervise the shared feature extractor, whereas the goat or horse target head used softmax outputs and focal categorical cross-entropy for mutually exclusive target-class prediction. Therefore, the source and target heads preserved their original annotation structures while sharing only the latent representation. When the dog dataset was used as the source or target, the corresponding head was optimized with binary cross-entropy for multi-label prediction; when goat or horse data were used, the corresponding head was optimized with categorical cross-entropy for single-label prediction.

#### Loss Formulation and Optimization

To make the dual-head optimization procedure explicit, the proposed framework was formulated as a shared feature extractor with domain-specific behavioral heads and a domain discriminator. Let $F_{\theta }(.)$ denote the shared temporal feature extractor, $C_{s}(.)$ the source behavioral classification head, $C_{t}(.)$ the target behavioral classification head, and $D_{\psi }\left(.\right)\;$the domain discriminator. For a source mini-batch $B_{s}=\{(x_{i}^{s},y_{i}^{s})\}$ and a target mini-batch $B_{t}=\{(x_{j}^{t},y_{j}^{t})\}$, the latent representations are given in Equation (5).

(5) $$z_{i}^{s}=F_{\theta }\left(x_{i}^{s}\right),\;{\;z}_{j}^{t}=F_{\theta }\left(x_{j}^{t}\right)$$

The output activation and classification loss of each behavioral head depend on the annotation structure of the corresponding domain. For multi-label data, the classifier uses independent sigmoid outputs; for single-label multi-class data, the classifier uses softmax outputs. Therefore, the same shared representation can be optimized under heterogeneous label structures without forcing the dog dataset into a single-label format or converting the goat and horse datasets into artificial multi-label targets.

For a multi-label head, let $y_{ik}\in \{0,1\}$ be the ground-truth indicator of class k for sample i, and let $p_{ik}=\sigma (a_{ik})$ be the corresponding sigmoid probability. The class-wise probability assigned to the true binary state is given in Equation (6).

(6) $${p^{*}}_{ik}=y_{ik}p_{ik}+(1-y_{ik})(1-p_{ik})$$

The focal binary cross-entropy loss used for multi-label prediction is given in Equation (7).

(7) $$L_{FBCE}=-\frac{1}{NK}\sum_{i=1}^{N}\sum_{k=1}^{K}\beta_{k}{{(1-p^{*}}_{ik})}^{\gamma_{BCE}}\;log({p^{*}}_{ik}+\varepsilon_{num})$$

where K is the number of unified behavior classes, $\beta_{k}$ is the optional class-balancing weight, $\gamma_{BCE}$ is the focal focusing parameter, and $\varepsilon_{num}$ is a small numerical constant used to avoid log(0). In the experiments, $\gamma_{BCE}=2.0$ was used for the source BCE term. When label smoothing was applied, binary labels were replaced by smoothed labels before computing the BCE term.

For a single-label multi-class head, let $y_{i}\in \{1,\ldots ,K\}$ denote the ground-truth class of sample i, and let $p_{i,c}$ be the softmax probability of class c. The focal categorical cross-entropy loss is given in Equation (8).

(8) $$L_{FCE}=-\frac{1}{N}\sum_{i=1}^{N}\omega_{yi}{{(1-p}_{i,yi})}^{\gamma_{CE}}\;log(p_{i,yi}+\varepsilon_{num})$$

where $\omega_{yi}$ is the class-balancing weight for the true class and $\gamma_{CE}$ is the focusing parameter. In the experiments, $\gamma_{CE}=1.5$ was used for the target CE term.

The supervised source classification loss $L_{src}$ is computed using either $L_{FBCE}$ or $L_{FCE}$, depending on the source-domain annotation structure. Similarly, the supervised target classification loss $L_{tgt}$ is computed using the loss function corresponding to the target-domain annotation structure. The combined classification loss is given in Equation (9).

(9) $$L_{cls}=L_{src}+{w_{tgt}L}_{tgt}$$

where $w_{tgt}$ controls the contribution of labeled target samples. In the main semi-supervised DANN experiments, $w_{tgt}=0.20\;$was used. In the fully unsupervised setting, $w_{tgt}=0$, and therefore no target classification gradient is used.

The domain discriminator receives the gradient-reversed latent representation and predicts whether each embedding originates from the source or target domain. Let $d_{i}\in \{0,1\}$ denote the domain label and $q_{i}=D_{\psi }({GRL}_{\lambda }(z_{i}))$ denote the predicted probability of the source domain. The binary domain-discrimination loss is given in Equation (10).

(10) $$L_{dom}=-\frac{1}{N_{s}+N_{t}}\sum_{i=1}^{N_{s}+N_{t}}\left[d_{i}\log\left(q_{i}+\varepsilon_{num}\right)+(1-d_{i})\log(1-q_{i}+\varepsilon_{num})\right]$$

The overall DANN optimization objective is given in Equation (11):

(11) $$\underset{\theta ,C_{s},C_{t}}{\min}\underset{\psi }{\max}[L_{cls}-\lambda (p)L_{dom}]$$

In implementation, this minimax behavior is obtained through the Gradient Reversal Layer (GRL). The GRL acts as an identity function during the forward pass but multiplies the gradient by −λ(p) during backpropagation, as shown in Equation (12).

(12) $${GRL}_{\lambda }\left(z\right)=z,\;\frac{\partial {GRL}_{\lambda }(z)}{\partial z}=-\lambda \left(p\right)I$$

Thus, the domain discriminator is trained to distinguish source and target embeddings, whereas the feature extractor receives the reversed domain gradient and is encouraged to learn species-invariant representations.

The GRL reversal coefficient was gradually increased using the standard sigmoid schedule in Equation (13).

(13) $$\lambda \left(p\right)=\lambda_{max}\left[\frac{2}{1+exp(-10p)}-1\right],\;p=\frac{e}{E}$$

where e is the current epoch, E is the total number of DANN training epochs, p∈[0,1] is the normalized training progress, and $\lambda_{max}\;$is the maximum adversarial weight. This schedule starts with a weak domain-alignment signal and progressively strengthens the adversarial objective as the behavioral classifier becomes more stable.

During the TL stage, only $L_{src}$ is optimized; target-domain samples, $L_{tgt}$, and the domain discriminator are not used. During the DANN stage, both source and target mini-batches are processed. The source loss updates the shared feature extractor and source head, while the target loss contributes only when $w_{tgt}>0.\;$In parallel, $L_{dom}$ is backpropagated through the GRL, encouraging the feature extractor to reduce behavioral classification error while making source and target embeddings less distinguishable.

### 2.5. Training Strategy and Experimental Design

The proposed framework was evaluated using six bidirectional source–target transfer pairs constructed across dog, goat, and horse datasets. For each experiment, one species was assigned as the source domain and another as the target domain to evaluate cross-species behavioral transferability under heterogeneous anatomical and behavioral conditions. The experimental pipeline consisted of three sequential training stages: TL, DANN, and Dynamic Pseudo Self-Training (D-PST). The dual-head source–target transfer scenarios evaluated in this study are summarized in Table 7.

During the TL stage, the feature extractor and classification heads were trained using only supervised source-domain data without adversarial adaptation. BCE was used for multi-label outputs with independent class probabilities, whereas CE was used for single-label multi-class outputs with mutually exclusive class probabilities. Dog data were treated as multi-label, while goat and horse data were treated as single-label multi-class. In the DANN stage, adversarial domain alignment was activated through the GRL model to reduce inter-species distribution discrepancies [22]. Semi-supervised target adaptation was performed by incorporating a limited number of labeled target samples into the optimization process using a target supervision coefficient ($w_{tgt}$). The primary experiments were conducted under semi-supervised conditions with $w_{tgt}=0.2$, while additional experiments were performed under sparse-label ($w_{tgt}=0.01$) and fully unsupervised ($w_{tgt}=0$) settings to analyze the effects of target supervision availability on adaptation stability and negative transfer behavior.

Labeled target samples used for semi-supervised adaptation were drawn only from the target-domain training partition and were never sampled from validation or test subjects. In the D-PST stage, 10 pseudo-labeled target samples per adaptation setting were used for target-head refinement, as indicated in Figure 1. Pseudo-labels were generated once after DANN using the best DANN checkpoint and only from the target-domain training partition. The term “dynamic” refers to the class-adaptive pseudo-label selection strategy: majority pseudo-label classes required a higher confidence threshold, whereas minority pseudo-label classes were allowed a lower threshold to reduce class-dominance bias. A single global confidence threshold was not used; instead, pseudo-label candidates were ranked within each predicted class according to posterior confidence, and the effective acceptance threshold varied according to predicted class frequency and class-level caps. Accepted pseudo-labels were ranked by confidence and limited by class-level caps. Pseudo-labels were not updated iteratively during D-PST; they remained fixed during refinement, and the adversarial domain loss was disabled. The complete experimental matrix, including transfer directions, model architectures, adaptation stages, and target supervision settings, is provided in Table 8.

All experiments were conducted under the same preprocessing, feature extraction, architecture, and training settings to ensure comparability across source–target pairs. Hyperparameters were not separately optimized for each transfer direction. Early stopping was based on validation loss, and the best validation checkpoint was used for final test evaluation. Fixed random seeds were used to ensure deterministic preprocessing and model initialization across experiments.

Models were trained using AdamW during the TL stage and Adam during the DANN and D-PST stages, as summarized in Table 6. Early stopping was applied based on validation loss to reduce overfitting during transfer learning. To ensure fair architectural comparison, identical preprocessing, augmentation, and training settings were applied across all evaluated feature extraction models. Experimental performance was analyzed across 72 transfer experiments covering multiple architectures, adaptation stages, and target supervision conditions.

### 2.6. K-Means Head Adaptation Strategy

To further investigate negative transfer behavior under severe domain discrepancy conditions, an additional lightweight target adaptation strategy based on K-Means clustering was evaluated after adversarial adaptation. In this stage, the feature extractor learned during DANN training was frozen, and only the target classification head was updated using cluster-guided target feature refinement. The objective of this procedure was to analyze whether species-invariant latent representations obtained through adversarial learning could be further stabilized through unsupervised target-space restructuring.

Initially, latent target representations extracted from the frozen feature extractor were grouped using K-Means clustering [45]. The number of clusters was selected according to the number of harmonized behavioral classes shared across species. Cluster assignments were subsequently used to initialize pseudo-target partitions within the latent feature space, enabling lightweight target-head adaptation without modifying the shared representation backbone. This strategy reduced computational complexity while preserving previously learned cross-species locomotor representations. To resolve the cluster-to-class alignment problem, cluster labels were not mapped manually. After K-Means clustering, each cluster was assigned to a behavioral class using the majority class among the sparse labeled target-training samples located in that cluster. If two clusters were assigned to the same class, the assignment was selected by maximizing the total number of matched labeled samples across clusters, following a Hungarian-style one-to-one matching rule. After this alignment, cluster assignments were converted into pseudo-target labels. The shared feature extractor was frozen, and only the target classification head was updated by minimizing the cross-entropy loss between the cluster-aligned pseudo-labels and the target-head predictions. No adversarial domain loss was used in this stage. Therefore, K-Means head adaptation was used only as a lightweight target-decision calibration step, not as a replacement for full semi-supervised DANN training.

The K-Means head adaptation stage was particularly designed to analyze the relationship between latent feature separability and transfer performance under sparse-label and fully unsupervised adaptation settings. In addition, the method enabled investigation of whether adversarially aligned behavioral representations formed biologically meaningful locomotor clusters independent of species identity. Experimental findings obtained from this stage were further evaluated using latent-space separability metrics and transfer performance analyses.

### 2.7. Evaluation Metrics and Statistical Analysis

Experimental performance was evaluated using Macro-F1, Micro-F1, Weighted-F1, precision, and recall metrics to analyze cross-species behavioral recognition performance under heterogeneous and imbalanced class distributions [46]. Since several behavioral categories contained substantially fewer samples than dominant locomotor classes, F1-based metrics were prioritized over overall accuracy to provide a more reliable assessment of minority-class recognition capability. Macro-F1 was used to evaluate balanced class-level performance, whereas Weighted-F1 and Micro-F1 were used to measure overall classification robustness under imbalanced behavioral distributions.

To evaluate latent representation quality and cross-species feature separability, Silhouette Score analysis was additionally performed on the learned feature embeddings [47]. This analysis was used to investigate whether adversarial adaptation improved species-invariant behavioral clustering within the shared latent representation space. Higher silhouette scores indicated improved intra-class compactness and inter-class separability across transferred behavioral representations.

Statistical significance analyses were conducted to compare architectural performance differences across transfer settings and adaptation stages. Pairwise comparisons between competing models were evaluated using the Wilcoxon signed-rank test [48], while multi-model performance comparisons were analyzed using the Friedman test [49] followed by Nemenyi post-hoc analysis [50]. Statistical analyses were performed to determine whether observed performance differences originated from consistent transfer improvements rather than random experimental variation. For the Wilcoxon signed-rank tests, each paired observation corresponded to the same source–target direction and model architecture evaluated under two different training stages. For the Friedman tests, repeated observations were formed across matched transfer settings, allowing comparison of training stages and model architectures under identical experimental conditions. Nemenyi post-hoc tests were then used to identify which stages or architectures differed significantly after the omnibus Friedman test.

## 3. Results

This section presents the experimental results obtained from the proposed cross-species behavioral representation learning framework. The results are organized according to the main experimental findings: the effect of representation-space selection, the contribution of domain-adversarial adaptation, source–target transfer performance, model architecture comparison, behavior-level recognition performance, sparse-label adaptation behavior, and statistical validation. Since the datasets contained heterogeneous and imbalanced behavioral distributions, Macro-F1 was used as the primary performance metric, while Precision–Recall Area Under the Curve (PR-AUC) was used as the primary complementary threshold-independent metric. Receiver Operating Characteristic Area Under the Curve (ROC-AUC) was retained only as a secondary descriptive metric for completeness because it may overestimate performance under severe class imbalance. Therefore, the interpretation of model performance was based primarily on Macro-F1 and PR-AUC rather than ROC-AUC. Because the train, validation, and test partitions were separated at the subject level, the reported results reflect performance on unseen animals rather than on windows from animals already observed during training. The results focus on quantifying whether adversarial representation learning can improve cross-species behavioral transferability while preserving biologically meaningful locomotor structures across heterogeneous animal species.

In addition to the main performance comparisons, the results include component-level ablation analyses to clarify whether the observed improvements were attributable to adversarial adaptation alone or to the combined preprocessing and representation-learning pipeline.

### 3.1. Preprocessing and Representation-Space Outcomes

The preprocessing pipeline reduced noisy and ambiguous training samples while preserving the test sets unchanged for unbiased evaluation. After ontology harmonization, segmentation, and cleaning, the datasets still showed heterogeneous class distributions, particularly in short-duration behaviors such as Shaking and high-intensity behaviors such as Running. Therefore, imbalance-aware augmentation and PCA-based representation stabilization were essential before cross-species adaptation.

Class balancing produced more stable training sets across species, although the dog dataset retained higher residual imbalance because of its multi-label annotation structure. The final balancing outcomes are summarized in Table 9.

Although class balancing reduced the imbalance in all datasets, residual imbalance remained more pronounced in the dog dataset, with a max/min ratio of 10.5:1 compared with 1.5:1 for the goat dataset and 1.8:1 for the horse dataset. This persistent imbalance is likely to be one of the structural factors contributing to the lower transfer performance observed in dog-targeted scenarios, particularly for the Shaking class.

The representation-space analysis showed that PCA strategy affected transferability. Goat-PCA produced the strongest average transfer performance in the preliminary logistic-regression analysis, followed by Horse-PCA, Dog-PCA, and Combined-PCA. In the Dog → Horse BiLSTM setting, PCA selection also influenced DANN performance, indicating that feature-space construction affected adversarial alignment quality. The mean transfer Macro-F1 values were averaged over the six source–target pairs. The deep-transfer comparison was conducted only for the Dog → Horse scenario using BiLSTM-Attention. These results are summarized in Table 10.

The PCA strategy analysis showed that Goat-fitted PCA achieved the highest mean transfer Macro-F1 in the logistic regression screening, followed by Horse-fitted PCA, whereas Joint PCA produced the lowest score. In the selected Dog → Horse BiLSTM-Attention setting, the effect of PCA depended on the learning strategy. Dog-fitted PCA performed better for TL only and K-Means head adaptation, while Goat-fitted PCA improved both unsupervised and semi-supervised DANN. This suggests that PCA spaces fitted on single-label datasets may provide a cleaner behavioral variance structure for adversarial adaptation. A separate dimensionality check under the Goat-fitted PCA setting showed that Macro-F1 increased from 0.564 with 5 components to 0.647 with 61 components, although the gain became smaller after 30 components. Therefore, the final experiments used a variance-preserving PCA setting to balance information retention and computational cost. In the final reported experiments, shared PCA refers to a single PCA transformation fitted only on the training partition and then applied consistently to the corresponding source and target validation/test partitions. It does not refer to separate PCA models fitted independently on source and target test data. The alternative Dog-fitted, Goat-fitted, Horse-fitted, and Joint PCA configurations in Table 10 were included as a representation-space sensitivity analysis. The large performance drop observed when removing shared PCA suggests that the deep temporal models benefited from a variance-stabilized input space before adversarial alignment, rather than being fully robust to raw heterogeneous IMU variability across species.

The Silhouette analysis (Table 11) further showed that the TL embedding produced the strongest behavior-level separation, while the DANN embedding yielded the lowest separation ratio, indicating improved domain alignment with a moderate reduction in class separability.

**Figure 3:**
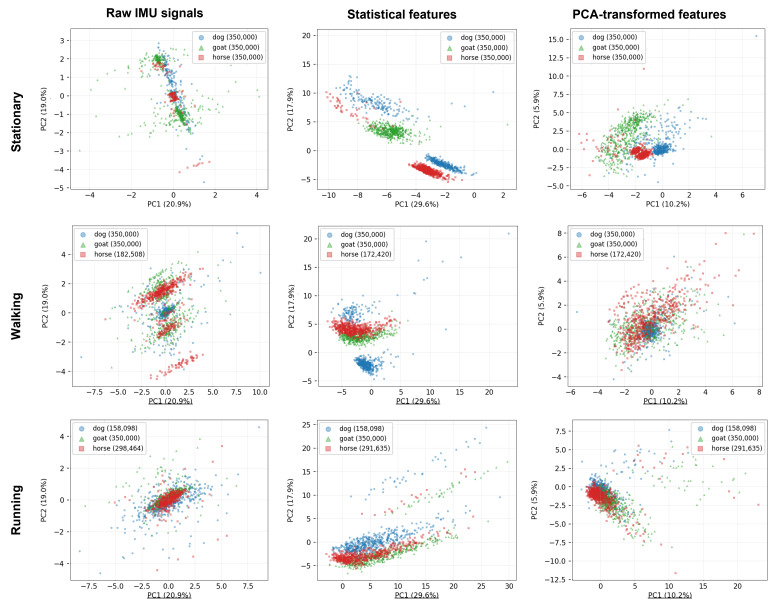
Evolution of representation spaces across preprocessing stages.

The feature-space visualizations supported these quantitative findings. As shown in Figure 3, compared with raw IMU representations, statistical features and PCA-transformed representations produced more structured and separable spaces. The contribution of each processing component is quantified later in Section 3.7.

### 3.2. Transfer Learning, DANN, and D-PST Performance

The overall phase comparison showed that DANN substantially improved cross-species transfer performance compared with direct transfer learning. Across the full experimental matrix, the average Macro-F1 increased from 0.345 in the TL phase to 0.711 in the DANN phase. This improvement indicates that adversarial feature alignment was effective in reducing inter-species distribution discrepancies.

**Figure 4:**
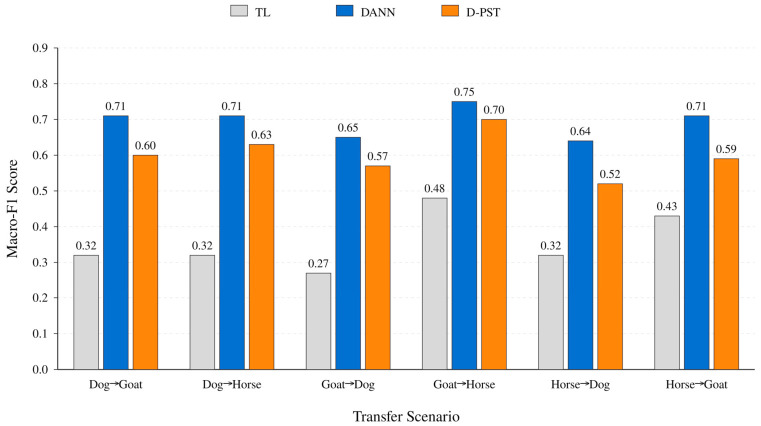
Source–target pair-based comparison of TL, DANN, and D-PST phases.

In contrast, the D-PST stage did not provide additional benefit after DANN. Although D-PST remained above the TL baseline in several cases, it reduced DANN performance in most experiments. Therefore, the best overall adaptation stage was DANN, not DANN followed by D-PST. The phase-level results are summarized in Table 12 and visualized in Figure 4.

Although D-PST remained above the TL baseline in some scenarios, its decrease relative to DANN indicates that pseudo-label errors accumulated after adversarial adaptation, particularly under class imbalance and behavior-level ambiguity. This degradation was interpreted together with the phase-level results in Table 12, the source–target trends in Figure 4, and the behavior-level F1 patterns reported in Section 3.4, rather than as evidence that D-PST provides a consistently beneficial refinement stage. The decline after D-PST was mainly interpreted as a confirmation-bias effect: pseudo-labels generated from already imperfect target predictions reinforced ambiguous decision boundaries, especially for short-duration or semantically overlapping behaviors such as Eating and Shaking. Although D-PST used class-adaptive confidence thresholding, confidence filtering alone was not sufficient to fully prevent pseudo-label noise under severe cross-species distribution shift. Therefore, D-PST is reported as a diagnostic negative result rather than as a consistently beneficial refinement stage.

### 3.3. Source–Target and Architecture-Level Results

The source–target analysis showed that the highest transfer performance was achieved in the Goat → Horse setting, with a Macro-F1 score of 0.747. This result suggests that transfer was more effective between species with compatible locomotor structures and single-label annotation schemes. In contrast, transfer settings targeting the dog dataset were more challenging because of its multi-label structure and higher residual imbalance. The source–target DANN results are presented in Table 13.

**Figure 5:**
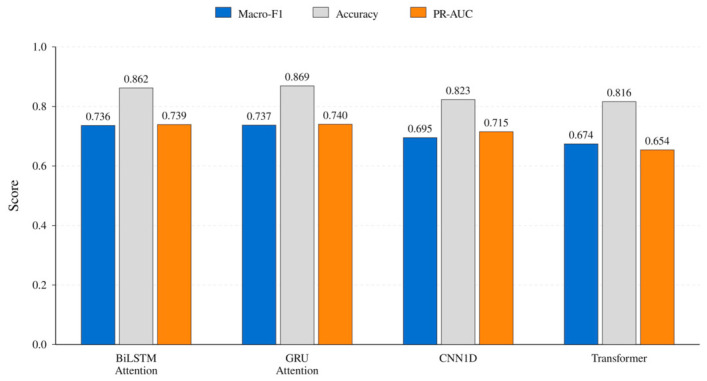
PR-AUC comparison of model performance under class imbalance.

Figure 4 shows that DANN consistently improved over transfer learning (TL) across all source–target directions, whereas Dynamic Pseudo Self-Training (D-PST) produced a systematic decline after Domain-Adversarial Neural Network (DANN) adaptation in most settings. The highest Mean Macro-F1 was obtained in the Goat → Horse transfer direction, indicating that the goat source domain provided the most transferable behavioral representation for the horse target domain. In contrast, dog-targeted transfers produced lower Macro-F1 and PR-AUC values, suggesting that the multi-label structure and residual class imbalance of the dog dataset made target adaptation more difficult. Accuracy and ROC-AUC remained high across all transfer directions, but PR-AUC better reflected the performance differences under class imbalance. Accordingly, Table 14 and Figure 5 were interpreted primarily in terms of PR-AUC, while ROC-AUC values were considered only secondary descriptive indicators.

Model-level results showed that attention-based recurrent architectures achieved the strongest overall performance. GRU-Attention obtained the highest average Macro-F1 score (0.737), followed very closely by BiLSTM-Attention (0.736). CNN1D performed moderately, whereas Transformer produced the lowest average Macro-F1 among the four architectures. These results indicate that recurrent attention-based architectures were more effective for extracting transferable temporal locomotor representations from wearable IMU data. The model-level results are summarized in Table 14 and Figure 5.

### 3.4. Behavior-Level Results

Behavior-level analysis showed that the proposed framework recognized rhythmic and temporally stable locomotor behaviors more successfully than short-duration or semantically overlapping behaviors. Trotting, Walking, and Stationary were the strongest classes, with several experiment–behavior pairs exceeding an F1 score of 0.90. In contrast, Shaking and Eating showed more variable performance because of their shorter duration, lower frequency, and stronger overlap with other low-mobility or head-related behaviors.

**Figure 6:**
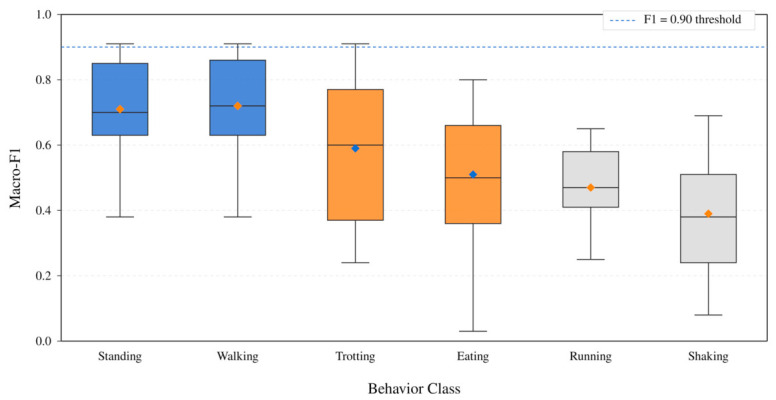
Behavior-level F1-score distribution in the DANN phase.

The behavior-level F1 distribution is shown in Figure 6, while the descriptive behavior-level statistics and high-performing experiment–behavior pairs are summarized in Table 15.

Stationary, Walking, and Trotting achieved the strongest class-level performance under DANN, with several behavior-specific transfer cases exceeding an F1 score of 0.90. In contrast, Running and Shaking remained the most challenging classes, reflecting the combined effects of class imbalance, short-duration movement patterns, and greater inter-species variability. The high-performing cases were mostly observed in goat- and horse-related transfer directions, suggesting that single-label target structures provided more stable class boundaries for adversarial adaptation.

These results indicate that longer-duration and rhythmically stable behaviors were more transferable across species, whereas short-duration or semantically overlapping behaviors were more sensitive to annotation mismatch, class imbalance, and inter-species biomechanical variability.

### 3.5. Sparse-Label and Unsupervised Adaptation Results

The target-label weight ablation revealed that fully unsupervised adversarial adaptation was unstable under strong inter-species discrepancy. In the Dog → Horse BiLSTM-Attention setting, the fully unsupervised condition ($w_{tgt}=0$) reduced Macro-F1 to 0.03, indicating severe negative transfer. However, even a very small target-label signal ($w_{tgt}=0.01$) increased Macro-F1 to 0.63, demonstrating that sparse target supervision was sufficient to stabilize adversarial adaptation.

The K-Means Head Adaptation strategy partially mitigated negative transfer under fully unsupervised target adaptation. Under the same unsupervised Dog → Horse setting, K-Means Head Adaptation achieved 0.40 Macro-F1, indicating a clear mitigation of fully unsupervised negative transfer compared with DANN. To address the diagnostic baseline limitation, a CORrelation ALignment (CORAL)-based feature alignment baseline was additionally evaluated in the same Dog → Horse BiLSTM-Attention setting. This baseline provided a non-adversarial discrepancy-reduction comparison and was used to determine whether covariance alignment could mitigate the negative transfer observed under fully unsupervised DANN. Table 16 summarizes the sparse target-supervision and unsupervised adaptation results in the representative Dog → Horse BiLSTM-Attention setting.

The fully unsupervised DANN result should be interpreted as a stress-test analysis rather than as evidence of universal failure across all source–target pairs and model architectures. The catastrophic negative transfer case (Macro-F1 = 0.03) was observed in the Dog → Horse setting using BiLSTM-Attention, which represents a difficult transfer scenario due to both inter-species biomechanical discrepancy and heterogeneous annotation structure. Therefore, we revised the interpretation to avoid overgeneralization. The result demonstrates that, under at least one severe cross-species shift, adversarial alignment without any target-label signal can collapse behavior-discriminative information. Broader evaluation of fully unsupervised DANN across all transfer pairs and architectures is left as a limitation and future work.

**Figure 7:**
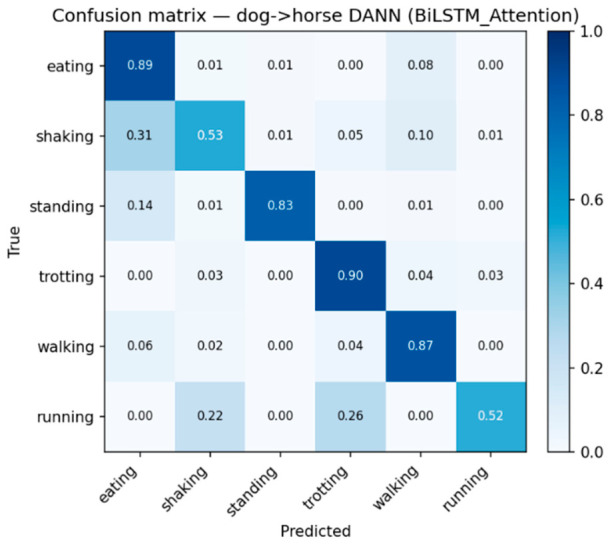
Confusion matrix for the representative Dog → Horse transfer scenario under DANN using the BiLSTM-Attention backbone.

As shown in Figure 7, the representative Dog → Horse DANN model preserved the main locomotor classes more effectively than short-duration or semantically overlapping behaviors. Most errors were concentrated between behavior pairs with similar inertial signatures, supporting the behavior-level interpretation reported in Section 3.4.

To clarify the scope of the baseline comparison, additional unsupervised target-adaptation baselines were evaluated in the Dog → Horse BiLSTM-Attention diagnostic setting. These baselines included a Conditional Domain Adversarial Network (CDAN)-style conditional adversarial adaptation variant, Pseudo-label-Assisted Domain-Adversarial Neural Network (PA-DANN), entropy minimization, nearest-centroid head adaptation, and K-Means head adaptation. This comparison was intended as a focused diagnostic analysis of whether alternative unsupervised adaptation strategies could mitigate the fully unsupervised DANN collapse, rather than as a comprehensive state-of-the-art benchmark across all source–target pairs and architectures. Fully unsupervised DANN and the adversarial variants remained below the TL-only zero-shot baseline, whereas nearest-centroid and K-Means head adaptation improved over TL-only by freezing the shared feature extractor and adapting only the target decision head. Cross-Species Knowledge Sharing and Preserving (CKSP) was not directly reimplemented because it follows a different multi-species joint-training protocol and was originally reported on different species, datasets, and evaluation conditions. Therefore, the present study does not claim superiority over CKSP or other non-reimplemented domain adaptation frameworks.

Fully unsupervised DANN showed severe negative transfer in the Dog → Horse setting, with Macro-F1 decreasing to 0.03. The target focal loss also increased from 1.41 to 4.35, indicating unstable target-domain adaptation. In contrast, adding a very small target-label signal, $w_{tgt}=0.01$, increased Macro-F1 to 0.63 and reduced the target focal loss from 1.27 to 0.71. Further increases in target supervision improved performance more gradually, reaching 0.72 at $w_{tgt}=0.20$ and 0.74 at $w_{tgt}=1.00$. Among fully unsupervised alternatives, K-Means head adaptation achieved the best Macro-F1 score of 0.40, but this value remained far below the semi-supervised DANN result. Therefore, K-Means head adaptation can reduce the severity of negative transfer but cannot fully compensate for the absence of target-label information. Other unsupervised variants, including the CDAN-style conditional adversarial variant, PA-DANN, and strong entropy minimization, were not retained in the table because they performed substantially worse in the diagnostic setting.

### 3.6. Statistical Validation

Statistical analyses confirmed that the observed performance differences were significant. The Wilcoxon signed-rank test was applied to paired model-stage results across identical source–target and architecture settings. The improvement from TL to DANN was statistically significant, and the decrease from DANN to D-PST was also significant. The Friedman test confirmed significant differences across training stages and model architectures. Nemenyi post-hoc comparisons showed that DANN significantly outperformed TL and D-PST, while attention-based recurrent architectures performed significantly better than the Transformer-based representation. The statistical results are summarized in Table 17.

The statistical analysis confirmed that the training stage had a significant effect on performance. All pairwise Wilcoxon comparisons among TL, DANN, and D-PST were significant, with W = 0.0 and *p* = 1.19 × 10^−7^. The Friedman test also showed a strong overall stage effect, supported by Kendall’s W = 1.00. The Nemenyi post hoc test confirmed significant differences among all stage pairs. Model architecture also had a significant effect. The post hoc analysis showed that the attention-based recurrent models differed significantly from Transformer, whereas their difference from CNN1D was not statistically significant. The very strong Kendall’s W and Wilcoxon results should be interpreted as consistency across the matched experimental configurations rather than as evidence of independent biological replication or absence of uncertainty. Specifically, W = 0.0 indicates that the paired stage comparisons showed the same directional ordering across the evaluated model-stage observations. Therefore, these statistical results should be interpreted together with the limitations related to missing confidence intervals, effect sizes, and repeated-seed variability.

### 3.7. Component-Level Ablation and Explainability

The ablation analysis demonstrated that the complete processing pipeline produced the most stable cross-species transfer behavior, with all components—ontology harmonization, noise cleaning, imbalance-aware augmentation, PCA-based feature stabilization, and DANN-based alignment—jointly contributing to the final performance rather than any single component alone. Removing the shared PCA transformation caused the largest drop, reducing Macro-F1 from 0.711 to 0.439, indicating that the shared PCA space was critical for reducing heterogeneous species-specific variance prior to adversarial alignment. Removing class balancing caused a substantial decrease particularly for minority classes, with F1 scores of Shaking and Running falling from 0.39 to 0.14 and from 0.48 to 0.18, respectively. Excluding minority-window reinforcement reduced the diversity of rare-class samples and lowered Macro-F1 to 0.519. Removing training-set cleaning produced the smallest decrease, suggesting that the DANN model was relatively robust to residual boundary noise compared with the effects of PCA and class balancing. The ablation scenarios are summarized in Table 18.

The ablation analysis was conducted in the Dog → Horse transfer setting using the BiLSTM-Attention backbone under semi-supervised DANN, because this setting represented a challenging cross-species transfer scenario and was used as a diagnostic case for component-level contribution analysis.

SHapley Additive exPlanations (SHAP) analysis further indicated that the learned models relied on physically meaningful motion-related features. The most influential predictors were associated with acceleration magnitude, gyroscope dynamics, temporal variability, and movement intensity. This supports the interpretation that the learned representations captured transferable locomotor information rather than dataset-specific artifacts. The averaged SHAP results are summarized in Table 19.

The SHAP analysis showed that gyroscope-derived zero-crossing features were the most consistently informative predictors across transfer settings. In particular, Gz_zcr and Gx_zcr ranked as the two most important features and appeared in the top-five feature list in 21 of the 24 scenarios. Gy_zcr and Gx_dom_ratio also showed high cross-scenario stability, indicating that rotational transition patterns and dominant-frequency structure were more discriminative than amplitude-based descriptors. Accelerometer-derived features were also relevant, especially Ax_zcr and Ay_zcr, but they appeared less consistently than gyroscope-based features. Overall, the results suggest that cross-species behavioral transfer was primarily driven by dynamic movement-transition characteristics rather than static signal magnitude alone.

Overall, the results show that semi-supervised domain-adversarial learning substantially improved cross-species behavioral transfer compared with direct transfer learning. The strongest results were obtained with attention-based recurrent architectures, particularly GRU-Attention and BiLSTM-Attention. DANN was consistently the most effective training phase, while D-PST reduced performance after adversarial adaptation. Sparse target supervision was critical for preventing negative transfer, and K-Means Head Adaptation provided a more stable but still limited unsupervised mitigation strategy.

## 4. Discussion

The findings of this study show that cross-species animal behavior recognition can be improved when wearable IMU data are modeled through transferable behavioral representations rather than through species-specific classification pipelines. The most important result was the substantial improvement obtained with domain-adversarial adaptation. Across the experimental matrix, the average Macro-F1 score increased from 0.345 in the TL phase to 0.711 in the DANN phase. This indicates that direct transfer learning was not sufficient to overcome anatomical, behavioral, and distributional differences between species, whereas adversarial feature alignment enabled the model to learn a more transferable latent representation.

These results support the assumption that different animal species may share partially transferable locomotor patterns, even though their morphology, gait dynamics, and annotation structures differ. In this sense, the biomimetic aspect of the framework lies in using biologically motivated locomotor similarity as a basis for representation transfer, rather than treating each species as a completely isolated classification problem.

### 4.1. Cross-Species Transferability and Adaptation Behavior

The comparison between TL, DANN, and D-PST provides a clear methodological conclusion. TL produced limited target-domain performance because the learned source representations remained biased toward the source species. DANN improved this limitation by reducing source–target distribution discrepancies through adversarial alignment. The consistent increase from TL to DANN indicates that cross-species behavioral recognition requires explicit domain adaptation rather than direct model reuse.

However, the results also show that adversarial adaptation has limits. In the Dog → Horse BiLSTM-Attention stress-test setting, the fully unsupervised condition $\left(w_{tgt}=0\right)$ reduced Macro-F1 to 0.03, indicating a severe risk of negative transfer under strong cross-species domain shift. This result should not be interpreted as a universal failure mode of unsupervised DANN across all transfer directions or architectures. Rather, it shows that, in this particularly difficult setting, adversarial alignment may remove behavior-discriminative information when no target-label signal is available. Adding a very small target-label contribution $\left(w_{tgt}=0.01\right)$ increased Macro-F1 to 0.63, suggesting that sparse target supervision can stabilize adaptation under severe domain shift.

A plausible explanation is that, in the fully unsupervised setting, the GRL optimizes domain confusion without any target-domain class anchor. Under strong anatomical and biomechanical differences, this can encourage the feature extractor to remove not only species-specific information but also behavior-discriminative structure. In this case, the model may satisfy the domain discriminator by over-aligning heterogeneous target features, leading to class-boundary collapse or majority-class-biased predictions. This interpretation is consistent with the sharp recovery observed when even a very small target-label signal was introduced.

The D-PST stage did not consistently improve DANN performance. Although pseudo-labeling can be useful in semi-supervised learning, the present results indicate that pseudo-label errors may accumulate after adversarial adaptation, especially for rare or ambiguous behaviors. This explains why D-PST reduced performance in most experiments. In contrast, K-Means Head Adaptation was more stable than fully unsupervised DANN under the tested unsupervised condition, but its performance remained substantially below semi-supervised DANN. This suggests that, when target labels are unavailable, adapting the decision head on clustered target embeddings may be safer than continuing full adversarial optimization.

### 4.2. Architecture, Behavior Classes, and Processing Pipeline

The architecture-level results showed that attention-based recurrent models produced the strongest transfer performance. GRU-Attention achieved the highest average Macro-F1 score, followed very closely by BiLSTM-Attention. This outcome is consistent with the sequential structure of IMU-based animal behavior data. Recurrent attention models can capture temporal dependencies and emphasize informative motion segments, which is particularly useful for rhythmic locomotor behaviors.

The weaker performance of Transformer and CNN1D can be interpreted in relation to the structure of the data. Transformer models generally require large and diverse datasets to learn stable global attention patterns, whereas CNN1D models are more limited in modeling long-range temporal dependencies. In contrast, GRU-Attention and BiLSTM-Attention provide a stronger inductive bias for medium-scale sequential sensor data. This explains why these architectures were more effective for cross-species transfer.

The behavior-level findings further support this interpretation. Trotting, Walking, and Stationary were recognized more reliably than Eating and Shaking. The better-performing classes have more stable temporal and biomechanical signatures, while Eating and Shaking are more variable, shorter in duration, or semantically overlapping with other behaviors. Eating may resemble low-mobility or head-down postures, and Shaking may be difficult to capture consistently within fixed windows. Therefore, transferability was not uniform across behaviors; it depended on temporal regularity, class frequency, and cross-species semantic consistency. Nevertheless, the operational ontology cannot completely eliminate sensor-level semantic mismatch. For example, dog Eating may co-occur with stationary posture or low head movement, whereas goat Eating often corresponds to sustained grazing-like head-down motion. This difference may partly explain the weaker and more variable Eating performance across transfer settings.

The ablation and preprocessing results also indicate that the final performance was produced by the complete pipeline rather than by a single component. Ontology harmonization reduced semantic mismatch between datasets. Noise cleaning removed ambiguous boundary samples. Class balancing improved minority behavior representation. PCA helped stabilize the feature space before adaptation. DANN then aligned source and target distributions in the learned representation space. Together, these components formed a coherent pipeline for improving cross-species transfer.

### 4.3. Implications and Explainability

The explainability results, summarized in Table 19, showed that the most influential features were related to gyroscope dynamics, zero-crossing behavior, acceleration magnitude, temporal variability, and movement intensity. The scenario-wise top five SHAP-ranked features across source–target transfer settings are provided in Supplementary Table S1. In particular, the frequent occurrence of Gz_zcr, Gx_zcr, Gy_zcr, and Gx_dom_ratio among the top-ranked SHAP features indicates that rotational transition patterns and dominant-frequency structures contributed strongly to cross-species behavioral discrimination.

The findings also have practical implications for animal monitoring systems. A model trained on one species can be partially transferred to another species when limited target labels are available. This can reduce annotation cost and support more scalable monitoring systems for animal welfare, livestock management, and comparative behavioral analysis. The strong performance of DANN under sparse supervision suggests that future systems do not need full target-domain annotation to become useful; carefully selected small target-label sets may be sufficient to guide adaptation.

### 4.4. Limitations

This study has several limitations. First, the experimental validation was limited to three animal species. Although subject-level partitioning prevented animal-level leakage, the goat dataset contained only five subjects, leaving one goat for validation and one goat for testing; therefore, goat-target transfer results should be interpreted cautiously and require confirmation on larger goat cohorts. Second, the datasets were collected in different original studies, and therefore differences in annotation protocol, animal population, recording context, and dataset balance may have affected transfer performance. Third, the dog dataset followed a multi-label structure, whereas the goat and horse datasets followed single-label structures, which made direct cross-species comparison more challenging. Relatedly, the six-class ontology intentionally reduced fine-grained behavioral specificity to create a shared cross-species label space; therefore, grouped behaviors such as playing, jumping, galloping, and running should not be interpreted as biologically identical across species. Fourth, the instability of fully unsupervised DANN was demonstrated only in the Dog → Horse BiLSTM-Attention stress-test setting; therefore, this result should be interpreted as evidence of potential negative transfer under severe domain shift rather than as a general conclusion for all transfer pairs and architectures. Fifth, the D-PST stage did not provide reliable improvement after DANN, suggesting that pseudo-label selection and confidence calibration require further methodological refinement. The ablation analysis was limited to major preprocessing and representation components in a single diagnostic transfer setting; additional sensitivity analyses for the BTSS weighting coefficient, GRL schedule, and focal-loss configuration should be performed in future work. The statistical analysis was based on matched model-stage comparisons rather than independent biological replicates. Therefore, the very strong Friedman and Wilcoxon results should be interpreted as evidence of consistent directional differences across the evaluated experimental matrix, not as proof of universally stable biological performance. Future work should report confidence intervals, effect sizes, repeated-seed variability, and broader replicate-based uncertainty estimates. In addition, the comparison with alternative domain adaptation methods was limited to a diagnostic Dog → Horse BiLSTM-Attention setting and did not constitute a full state-of-the-art benchmark across all transfer pairs and architectures. Therefore, future work should include broader comparisons with recent domain adaptation and self-supervised time-series representation methods, including margin disparity discrepancy (MDD), minimum class confusion (MCC), CORAL, maximum mean discrepancy (MMD)-based approaches, and modern self-supervised time-series encoders. The SHAP analysis was used only as a post-hoc feature-level interpretability tool and does not provide direct biological validation of the learned representations. Future work should include class-specific SHAP analysis, confusion matrices, error-case inspection, and broader hyperparameter sensitivity analyses to better assess the robustness of the reported performance gains. Finally, the proposed framework should be externally validated on additional species, sensor placements, and real-world deployment conditions before being considered a general-purpose animal behavior monitoring solution.

Overall, the results show that semi-supervised domain-adversarial representation learning can substantially improve cross-species behavioral transfer from wearable IMU data. The strongest performance was obtained when adversarial alignment was combined with sparse target supervision and attention-based recurrent feature extraction. These findings suggest that transferable locomotor representations can be learned across heterogeneous animal species, provided that semantic label harmonization, imbalance-aware preprocessing, and adaptation stability are carefully addressed.

## 5. Conclusions

This study presented a semi-supervised cross-species behavioral representation learning framework for transferable animal activity recognition using wearable IMU data from dogs, goats, and horses. The proposed approach combined behavioral ontology harmonization, imbalance-aware preprocessing, PCA-based representation stabilization, temporal deep feature extraction, and domain-adversarial adaptation within a unified learning pipeline. The main objective was to determine whether shared locomotor structures could be learned across anatomically and behaviorally heterogeneous species while reducing dependence on large-scale labeled target datasets.

The experimental results demonstrated that domain-adversarial adaptation substantially improved cross-species transfer performance. Across the evaluated transfer settings, the average Macro-F1 score increased from 0.345 in the direct transfer learning phase to 0.711 in the DANN phase. Attention-based recurrent architectures achieved the strongest performance, with GRU-Attention and BiLSTM-Attention outperforming Transformer and CNN1D models. The highest transfer performance was obtained in the Goat → Horse setting, suggesting that behavioral transfer is more effective when target and source domains share compatible locomotor structures and annotation schemes. Behavior-level results further showed that rhythmic and temporally stable behaviors, such as Trotting, Walking, and Stationary, were more transferable than short-duration or semantically overlapping behaviors.

The Dog → Horse BiLSTM-Attention stress-test analysis showed that fully unsupervised adversarial adaptation may become unstable under severe inter-species domain shift. In this setting, the absence of target supervision reduced Macro-F1 to 0.03, whereas a very small target-label contribution increased Macro-F1 to 0.63. Therefore, this result is interpreted as evidence of a potential negative-transfer risk rather than as a universal conclusion for all transfer pairs and architectures. K-Means Head Adaptation provided a more stable but limited mitigation strategy in the same unsupervised setting. Broader evaluation of fully unsupervised DANN across all transfer directions and model architectures remains an important direction for future work.

Overall, the findings support the feasibility of learning biologically meaningful and transferable behavioral representations from wearable sensor data across animal species. The proposed framework offers a scalable direction for animal behavior monitoring systems by reducing the need for extensive species-specific annotation and model redesign. Future work should extend the framework to additional species, sensor placements, and real-world deployment conditions. Further improvements may also be achieved by integrating confidence-aware pseudo-labeling, adaptive ontology refinement, stronger target-domain calibration, and multimodal sensing sources such as video, GPS, or physiological signals.

The results should therefore be interpreted as evidence for semi-supervised cross-species transferability under controlled public-dataset conditions, rather than as proof of fully unsupervised generalization across all animal species and sensing environments.

## Figures and Tables

**Table 1:** Technical characteristics of the datasets used in the study.

Characteristic	Dog Dataset *	Goat Dataset	Horse Dataset
Reference	Kumpulainen et al. [11]	Kamminga et al. [34]	Kamminga et al. [12]
Number of subjects	45 dogs from 27 breeds	5 goats, including 3 Pygmy and 2 larger goats	18 animals, including horses and ponies
Sensor device	ActiGraph GT9X Link	ProMove-mini, Inertia Tech.	Gulf Coast Data Concepts HAM
Sensor placement	Neck	Neck	Neck
Sensor modality	Tri-axial accelerometer and gyroscope	Tri-axial accelerometer and gyroscope	Tri-axial accelerometer and gyroscope
Recorded sensor channels	ANeck_x, ANeck_y, ANeck_z, GNeck_x, GNeck_y, GNeck_z	ANeck_x, ANeck_y, ANeck_z, GNeck_x, GNeck_y, GNeck_z	ANeck_x, ANeck_y, ANeck_z, GNeck_x, GNeck_y, GNeck_z
Sampling frequency	100 Hz	100 Hz	100 Hz
Classification setting	Multi-label classification	Single-label multi-class classification	Single-label multi-class classification

* In the dog dataset, each sample may be associated with multiple behavior annotations, whereas the goat and horse datasets assign a single behavior class to each sample.

**Table 2:** Unified six-class behavior ontology and mapping of original dataset labels.

Unified Target Class	Mapped Original Labels	Operational Definition
Eating (feeding-related behavior)	Eating, drinking, breastfeeding, grazing	Feeding- or drinking-related behaviors characterized by repetitive head-down movements during food or water intake, including grazing and nursing-related activity.
Shaking (grooming-like behavior)	Shaking, headshake, scratch biting	Rapid whole-body or head movements associated with shaking, irritation relief, grooming-like scratching, or biting-related self-directed actions.
Stationary (postural behavior)	Standing, sitting, panting, rubbing, scared, lying chest, lying	Low-mobility postural states in which the animal remains largely stationary, including standing, sitting, lying, and other non-locomotor behaviors.
Walking (low-intensity locomotion)	Walking, walking-natural, walking-rider, pacing, sniffing, carrying object, synchronization	Low-speed locomotion involving regular forward movement, including natural or ridden walking and slow exploratory or socially synchronized movement.
Trotting (moderate-intensity locomotion)	Trotting, trotting-natural, trotting-rider, climbing up, climbing down	A moderate-speed rhythmic locomotion pattern, faster than walking but slower than running, including diagonal gait patterns and inclined movement.
Running (high-intensity locomotion)	Galloping, running, galloping-natural, galloping-rider, jumping, playing	High-speed or high-intensity locomotor activity involving rapid acceleration, asymmetric gait patterns, jumping, galloping, or play-related explosive movement.

**Table 3:** Window-level class distribution after ontology harmonization.

Unified Class	Dataset	Raw Sample Count *	Raw Proportion (%)	Windowed Segment Count	Windowed Proportion (%)	Change (p.p.)
Eating	Dog	377,480	4.6	287,233	5.8	+1.2
Shaking		42,047	0.5	36,737	0.7	+0.2
Stationary		4,755,740	58.2	2,274,018	45.7	−12.5
Walking		764,540	9.4	637,491	12.8	+3.4
Trotting		2,013,960	24.6	1,579,090	31.7	+7.1
Running		216,674	2.7	162,909	3.3	+0.6
Total		8,170,441	100.0	4,977,478	100.0	
						
Eating	Goat	23,854,911	36.4	2,177,163	28.7	−7.7
Shaking		564,936	0.9	356,313	4.7	+3.8
Stationary		26,058,818	39.8	2,254,190	29.8	−10.0
Walking		1,430,779	2.2	1,014,163	13.4	+11.2
Trotting		12,801,506	19.5	1,130,164	14.9	−4.6
Running		794,419	1.2	643,063	8.5	+7.3
Total		65,505,369	100.0	7,575,056	100.0	
						
Eating	Horse	2,086,567	19.5	176,852	10.2	−9.3
Shaking		156,139	1.5	100,597	5.8	+4.3
Stationary		623,823	5.8	533,174	30.6	+24.8
Walking		2,910,088	27.3	242,916	14.0	−13.3
Trotting		4,435,754	41.5	365,613	21.0	−20.5
Running		464,204	4.3	321,359	18.5	+14.2
Total		10,676,575	100.0	1,740,511	100.0	

* Raw sample count refers to the number of time-point-level sensor observations before windowing, whereas windowed segment count refers to the number of samples obtained after temporal segmentation. Change is reported in percentage points, calculated as the difference between the windowed and raw class proportions. For the dog dataset, which is multi-label, the class counts refer to behavior-label assignments rather than mutually exclusive class instances.

**Table 4:** Exact subject-level train/validation/test allocation.

Dataset	Total Subjects	Training Subjects	Validation Subjects	Test Subjects
Dog	45	31	7	7
Goat	5	3	1	1
Horse	18	12	3	3

**Table 5:** Data-cleaning statistics after ENN and Tomek Links filtering.

Dataset	Initial Training Windows	Removed Training Windows *	Final Training Windows	Removal Rate (%)
Dog	4,960,199	706,500	4,253,699	14.2
Goat	4,158,772	301,345	3,857,427	7.2
Horse	1,265,706	6752	1,258,954	0.5

* Removed training windows refer to samples excluded through the sequential application of Sparse Random Projection (SRP)-assisted Mahalanobis outlier detection, Edited Nearest Neighbors, and Tomek Links. The test set was not subjected to this cleaning procedure in order to preserve an unbiased evaluation protocol.

**Table 6:** Common training configuration and hyperparameters used across all models.

Component	Hyperparameter	Setting
Optimization	Optimizer	AdamW for Transfer Learning (TL); Adam for DANN and D-PST
	Learning rate	5 × 10^−4^ for TL; 1 × 10^−4^ for DANN; 1 × 10^−5^ for D-PST
	Learning-rate schedule	Linear warm-up for TL, followed by cosine annealing in all stages
Training setup	Batch size	128
	Maximum epochs	120 for TL; 60 for DANN; 10 for D-PST
	Early stopping patience	20 for TL; 7 for DANN; 7 for D-PST
Regularization	Dropout rate	0.5
	Weight decay	5 × 10^−4^ for TL
	Gradient clipping	max_norm = 1.0
	Input noise	Gaussian noise with σ = 0.3 during TL
Loss configuration	Label smoothing	ε = 0.05 for source BCE
	Focal-loss parameter	$\gamma_{BCE}$ = 2.0; $\gamma_{CE}$ = 1.5
	Entropy weight	w_ent = 0.01
	Rare-class loss weight	w_rare = 0.5
BTSS weighting	Transferability coefficient	α = 0.7

**Table 7:** Dual-head source–target transfer scenarios.

Transfer Setting	Source → Target Pair	Source Head	Target Head
Multi-label to single-label multi-class	Dog → Goat; Dog → Horse	Sigmoid + focal BCE	Softmax + focal CE
Single-label multi-class to multi-label	Goat → Dog; Horse → Dog	Softmax + focal CE	Sigmoid + focal BCE
Single-label multi-class to single-label multi-class	Goat → Horse; Horse → Goat	Softmax + focal CE	Softmax + focal CE

**Table 8:** Experimental matrix of source–target transfer directions.

Source Domain	Target Domain	Transfer Direction *
Dog	Goat	Multi-label to single-label multi-class
Dog	Horse	Multi-label to single-label multi-class
Goat	Dog	Single-label multi-class to multi-label
Goat	Horse	Single-label multi-class to single-label multi-class
Horse	Dog	Single-label multi-class to multi-label
Horse	Goat	Single-label multi-class to single-label multi-class

* All source–target transfer settings were evaluated using four backbone architectures: BiLSTM-Attention, GRU-Attention, CNN1D, and Transformer. Each model was trained and evaluated across three sequential stages: TL, DANN, and D-PST. Accordingly, the experimental matrix comprised six transfer directions, four model architectures, and three training stages, yielding 72 model-stage evaluations in total.

**Table 9:** Final training-set class distribution after class balancing.

Dataset	Balancing Strategy	Final Training Windows	Target Size	Eating	Shaking	Stationary	Walking	Trotting	Running	Max/Min Ratio
Dog *	Proto-selection + MLSMOTE + ENN	992,522	376,974 per label	155,288	39,318	412,885	324,887	161,636	95,474	10.5:1
Goat	cWGAN-GP + K-Means	approximately 3,315,000	593,045	593,045	384,661	593,045	558,156	593,045	593,045	1.5:1
Horse	ENN + cWGAN-GP + K-Means	824,887	163,869	93,501	109,243	163,869	163,869	130,536	163,869	1.8:1

* For the dog dataset, class counts indicate label-level assignments because the dataset is multi-label. Therefore, class counts do not sum to the number of final training windows. For the goat and horse datasets, which follow a single-label multi-class setting, class counts correspond to mutually exclusive class instances. The max/min ratio was calculated using the largest and smallest class counts after balancing.

**Table 10:** PCA strategy and representation-space evaluation.

PCA Fitting Strategy	Retained Components	Mean Transfer Macro-F1, Logistic Regression (LR), Six Pairs	TL Only	DANN, Unsupervised	K-Means Head	DANN, Semi-Supervised
Dog-fitted PCA	59	0.265	**0.33**	0.03	**0.40**	0.72
Goat-fitted PCA	61	0.307 *	0.31	**0.14**	0.32	**0.80**
Horse-fitted PCA	61	0.301	—	—	—	—
Joint PCA	62	0.263	—	—	—	—

* Bold values indicate the best result within each comparison.

**Table 11:** Representation-space quality across the data processing pipeline.

Representation Stage	Dimensionality	Domain Silhouette	Behavior Silhouette	Separation Ratio
Raw IMU signals	6	0.258	−0.108	1.563
Statistical features	98	0.028	0.011	0.691
PCA-transformed features	59	0.062	0.063	0.670
TL embedding	512	**0.010**	0.233 *	0.623
DANN embedding	512	0.052	0.193	**0.588**

* Bold values indicate the most favorable value for each metric.

**Table 12:** Phase-level average performance across TL, DANN, and D-PST.

Training Stage	Mean Macro-F1	Mean Accuracy
TL	0.345	0.490
DANN	0.711 *	**0.846**
DANN + D-PST	0.643	0.790

* Bold values indicate the best-performing stage.

**Table 13:** Source–target pair-based DANN performance averaged across model architectures.

Source → Target	Mean Macro-F1	Accuracy	ROC-AUC	PR-AUC
Dog → Goat	0.715	0.884	0.962	0.731
Dog → Horse	0.714	0.834	0.948	0.745
Goat → Dog	0.645	0.798	0.924	0.634
Goat → Horse	**0.747**	0.863	0.957	**0.781**
Horse → Dog	0.641	0.809	0.930	0.633
Horse → Goat	0.713	**0.886**	**0.964**	0.748

Values represent the mean performance of the DANN stage across the evaluated model architectures. Bold values indicate the best result for each metric. To further clarify the effect of heterogeneous label structures, transfer settings were descriptively grouped according to the target-domain annotation format. Single-label-to-single-label transfers, namely Goat → Horse and Horse → Goat, achieved a mean Macro-F1 of 0.730. Transfers targeting the multi-label dog dataset, namely Goat → Dog and Horse → Dog, achieved a lower mean Macro-F1 of 0.643. Dog-source transfers to single-label targets, namely Dog → Goat and Dog → Horse, achieved a mean Macro-F1 of 0.715. This pattern suggests that the main label-structure difficulty occurred when the target domain followed a multi-label annotation scheme. However, this comparison should be interpreted as a descriptive group-level analysis rather than as a fully controlled relabeling ablation.

**Table 14:** Model-architecture performance comparison with PR-AUC emphasized under class imbalance.

Model Architecture	Mean Macro-F1	Accuracy	ROC-AUC	PR-AUC
BiLSTM-Attention	0.736	0.862	**0.958**	0.739
GRU-Attention	**0.737**	**0.869**	0.955	**0.740**
CNN1D	0.695	0.823	0.946	0.715
Transformer	0.674	0.816	0.930	0.654

The difference between GRU-Attention and BiLSTM-Attention was negligible, whereas both recurrent attention-based models clearly outperformed CNN1D and Transformer in Macro-F1. Therefore, the main architectural conclusion is not that one recurrent model is dominant, but that attention-based recurrent temporal modeling is more suitable for transferable IMU representations in this setting.

**Table 15:** Behavior-level DANN performance summary.

Unified Target Class	Mean Class F1	F1 Range	High-Performing Transfer Cases, F1 > 0.90
Eating	0.655	0.034–0.899	None
Shaking	0.393	0.108–0.665	None
Stationary	0.828	0.541–0.923	Dog → Goat, BiLSTM, 0.913; Horse → Goat, CNN1D, 0.911; Horse → Goat, GRU, 0.902
Walking	0.790	0.032–0.922	Dog → Goat, GRU, 0.917; Dog → Horse, GRU, 0.908; Goat → Horse, BiLSTM, 0.906; Goat → Horse, GRU, 0.901
Trotting	0.742	0.381–0.920	Goat → Horse, GRU, 0.920; Goat → Horse, BiLSTM, 0.912
Running	0.448	0.106–0.655	None

**Table 16:** Sparse target-supervision and unsupervised adaptation ablation in the Dog → Horse scenario using BiLSTM-Attention.

Analysis	Method/Setting	$w_{tgt}$	Macro-F1	Accuracy
Target-supervision ablation	DANN, unsupervised	0.00	0.03	0.20
	DANN, sparse supervision	0.01	0.63	—
	DANN, low supervision	0.10	≈0.70	—
	DANN, semi-supervised	0.20	0.72	0.85
	DANN, full target signal	1.00	0.74 *	**0.86**
Unsupervised comparison	TL only, zero-shot	0.00	0.33	—
	Nearest centroid head	0.00	0.39	—
	K-Means head adaptation	0.00	**0.40**	—
	CORAL feature alignment	0.00	0.33	0.45

* The unsupervised comparison includes only the strongest non-DANN baselines. The K-Means head adaptation result should be interpreted as a partial mitigation of negative transfer under fully unsupervised conditions, not as a replacement for semi-supervised DANN. Bold values indicate the best result within each analysis group.

**Table 17:** Statistical validation of training-stage and model-architecture effects.

Analysis Factor	Comparison	Statistical Test	Test Statistic	*p*-Value	Effect Size	Macro-F1 Difference	95% Bootstrap CI	Interpretation
Training stage	TL vs. DANN vs. D-PST	Friedman	χ^2^ = 46.08; Kendall’s W = 0.96	9.84 × 10^−11^ < 0.001	Kendall’s W = 0.96	+0.351 (max gap, DANN−TL)	[+0.324, +0.377]	Significant stage effect
Training stage	DANN − TL	Wilcoxon signed-rank *	W = 0.0 for all comparisons	1.19 × 10^−7^ < 0.001	r_rb =1.00	+0.351	[+0.324, +0.377]	All stage pairs differed significantly
Training stage	DANN − D-PST	Wilcoxon signed-rank *	W = 2.0	≤ 0.0015	r_rb = 0.987 (23/1)	+0.053	[+0.027, +0.087]	TL, DANN, and D-PST differed significantly
Training stage	D-PST − TL	Wilcoxon signed-rank *	W = 0.0	1.19 × 10^−7^ ≤ 0.001	r_rb =1.00	+0.297	[+0.259, +0.334]	D -PST significantly improves over the TL baseline
Model architecture	Four architectures	Friedman	χ^2^ = 11.80	0.0081 ≤ 0.01	Kendall’s W = 0.66	+0.063	[+0.034, +0.098]	Significant architecture effect
Model architecture, post hoc	Recurrent attention models vs. Transformer/CNN1D	Nemenyi	Δrank = 2.17	0.037 / 0.279	r_rb = 1.00	+0.062	[+0.035, +0.091]	Recurrent attention models differed from Transformer, but not from CNN1D

* The Wilcoxon signed-rank tests were conducted over 24 paired experiments. Significance was assessed at α = 0.05.

**Table 18:** Component-level ablation analysis of the preprocessing and representation pipeline in the Dog → Horse transfer setting using BiLSTM-Attention under semi-supervised DANN.

Scenario	Configuration	Removed Component	Macro-F1	ΔMacro-F1
S0	Full pipeline	None	0.711	—
S1	Without class balancing	Class balancing	0.469	−0.241
S2	Without shared PCA	Shared PCA transformation	0.439	−0.272
S3	Without training-set cleaning	Training-set cleaning	0.586	−0.124
S4	Without minority-window reinforcement	Minority-window reinforcement	0.519	−0.191

**Table 19:** Overall SHAP feature importance and cross-scenario stability across 24 transfer settings.

Rank	Feature	Feature Type	Mean SHAP Importance	Standard Deviation	Top-5 Occurrence, *n*/24
1	Gz_zcr	Gyroscope, zero-crossing rate	1.016	0.436	21
2	Gx_zcr	Gyroscope, zero-crossing rate	0.990	0.422	21
3	Gx_dom_ratio	Gyroscope, dominant-frequency ratio	0.984	0.421	18
4	Gy_zcr	Gyroscope, zero-crossing rate	0.978	0.409	19
5	Ax_zcr	Accelerometer, zero-crossing rate	0.966	0.401	15
6	Gz_dom_ratio	Gyroscope, dominant-frequency ratio	0.942	0.389	4
7	Ay_zcr	Accelerometer, zero-crossing rate	0.942	0.387	8
8	Gy_dom_ratio	Gyroscope, dominant-frequency ratio	0.939	0.386	3
9	svm_acc_mean	Signal-magnitude feature	0.918	0.411	2
10	Az_zcr	Accelerometer, zero-crossing rate	0.889	0.366	1
11	Az_entropy	Accelerometer, entropy	0.870	0.357	0
12	Az_band35	Accelerometer, spectral-band energy	0.864	0.413	2
13	Gz_rms	Gyroscope, RMS amplitude	0.859	0.417	2
14	Ay_rms	Accelerometer, RMS amplitude	0.851	0.431	1
15	Gz_entropy	Gyroscope, entropy	0.846	0.341	0

## Data Availability

The datasets analyzed in this study are publicly available through the original studies. The dog dataset is available through Kumpulainen et al. [11], https://doi.org/10.1016/j.applanim.2021.105393. The goat dataset is available through Kamminga et al. [34], https://doi.org/10.1145/3191747. The horse dataset is available through Kamminga et al. [12], https://doi.org/10.3390/data4040131. No new animal dataset was generated during this study. Processed data tables, ontology mappings, and experimental outputs are available from the corresponding author upon reasonable request.

## Supplementary Materials

The following supporting information can be downloaded at: https://www.mdpi.com/article/10.3390/biomimetics11070496/s1, Figure S1: Raw-label-level JSD distance matrix between dog and goat behavior labels; Figure S2: Raw-label-level JSD distance matrix between dog and horse behavior labels; Figure S3: Raw-label-level JSD distance matrix between goat and horse behavior labels; Figure S4: Unified ontology-class-level mean JSD distance matrix for the Dog → Goat transfer setting; Figure S5: Unified ontology-class-level mean JSD distance matrix for the Dog → Horse transfer setting; Figure S6: Unified ontology-class-level mean JSD distance matrix for the Goat → Horse transfer setting; Table S1: Scenario-wise top five SHAP-ranked features across source–target transfer settings.

## Abbreviations

The following abbreviations are used in this manuscript:

BCE	Binary Cross-Entropy
BiLSTM	Bidirectional Long Short-Term Memory
BTSS	Behavioral Transferability Similarity Score
CDAN	Conditional Domain Adversarial Network
CE	Categorical Cross-Entropy
CKSP	Cross-Species Knowledge Sharing and Preserving
CNN	Convolutional Neural Network
CNN1D	One-Dimensional Convolutional Neural Network
CORAL	CORrelation ALignment
cWGAN-GP	Conditional Wasserstein Generative Adversarial Network with Gradient Penalty
DANN	Domain-Adversarial Neural Network
D-PST	Dynamic Pseudo Self-Training
ENN	Edited Nearest Neighbors
GAN	Generative Adversarial Network
GRL	Gradient Reversal Layer
GRU	Gated Recurrent Unit
IMU	Inertial Measurement Unit
JSD	Jensen–Shannon Divergence
KL	Kullback–Leibler
LR	Logistic Regression
LSTM	Long Short-Term Memory
MCC	Minimum Class Confusion
MDD	Margin Disparity Discrepancy
MLSMOTE	MLSMOTE
MMD	Maximum Mean Discrepancy
PA-DANN	Pseudo-label-Assisted Domain-Adversarial Neural Network
PCA	Principal Component Analysis
p.p.	percentage points
PR-AUC	Precision–Recall Area Under the Curve
ROC-AUC	Receiver Operating Characteristic Area Under the Curve
SHAP	SHapley Additive exPlanations
SRP	Sparse Random Projection
TL	Transfer Learning
$w_{tgt}\;$	Target supervision coefficient
