# Supplementary material for: Cross-Species Behavioral Representation Learning Using Domain-Adversarial Adaptation on Wearable IMU Signals
Source: Biomimetics (Basel). 2026 Jul 15;11(7):496. doi: 10.3390/biomimetics11070496 (PMC13406206; doi:10.3390/biomimetics11070496)
Supplement: Supplementary file 1 [file biomimetics-11-00496-s001.zip › biomimetics-4380266-supplementary.pdf]

Figure S1. Raw-label-level JSD distance matrix between dog and goat behavior labels.

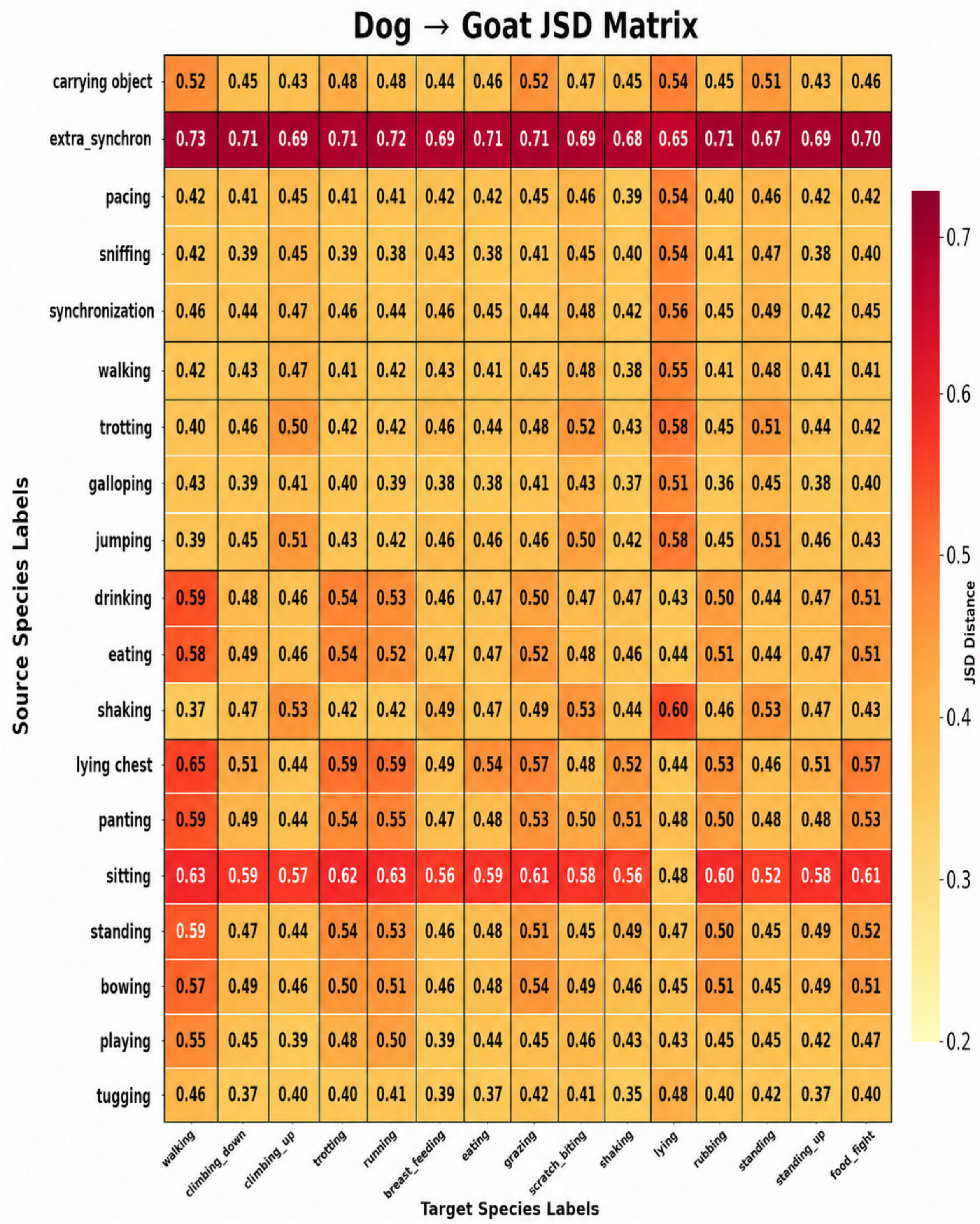

Figure S2. Raw-label-level JSD distance matrix between dog and horse behavior labels.

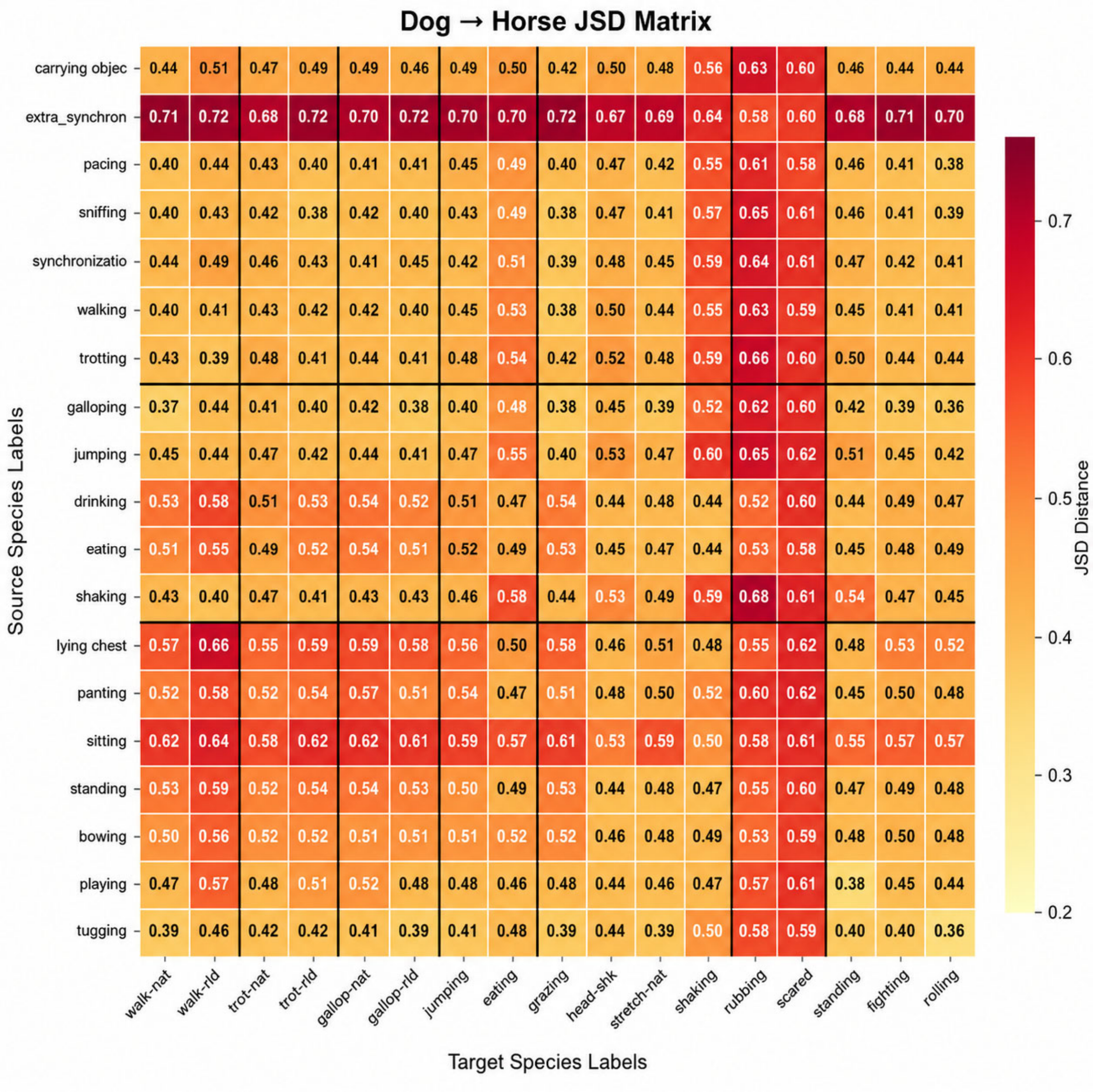

Figure S3. Raw-label-level JSD distance matrix between goat and horse behavior labels.

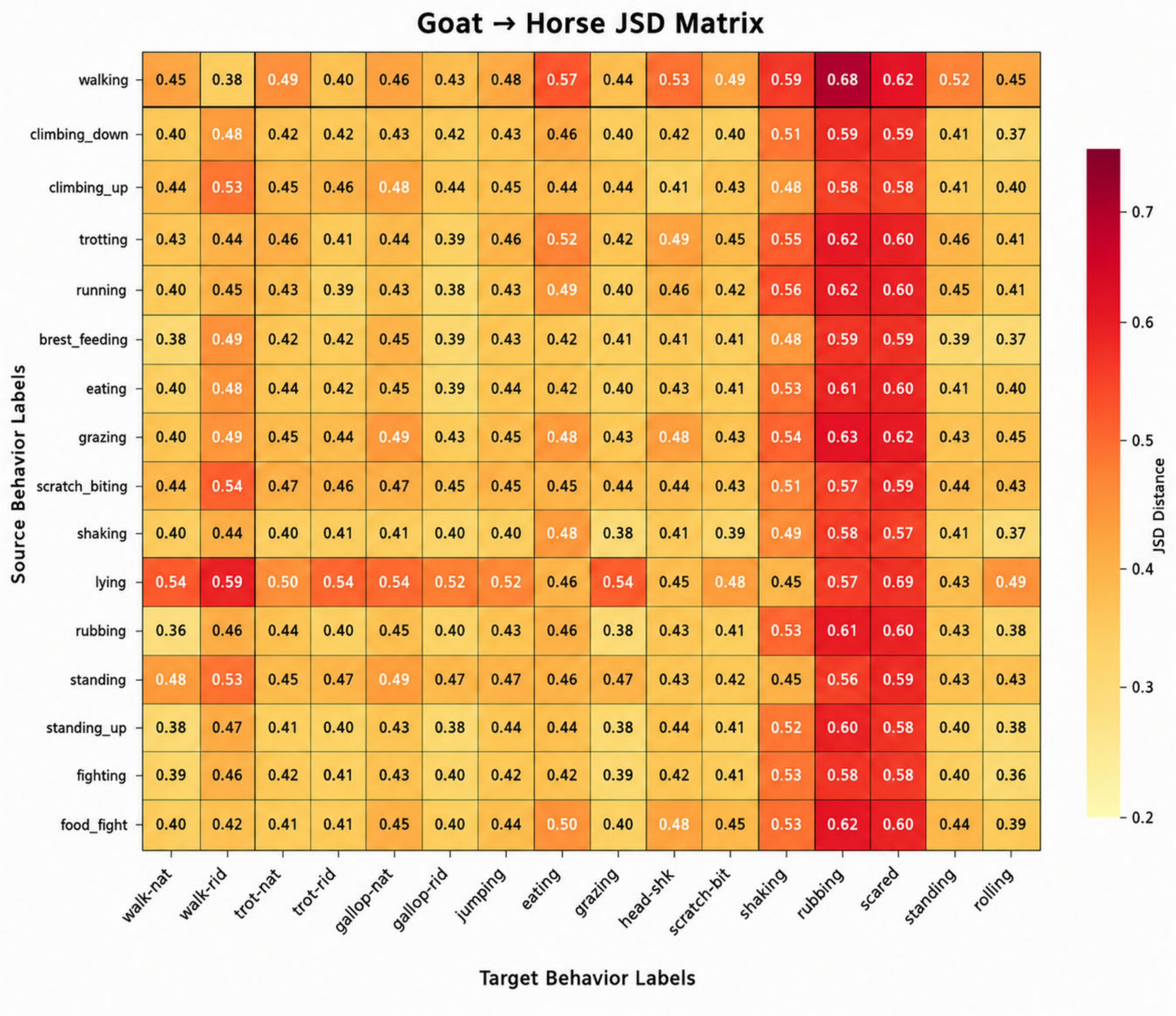

Figure S4. Unified ontology-class-level mean JSD distance matrix for the Dog → Goat transfer setting.

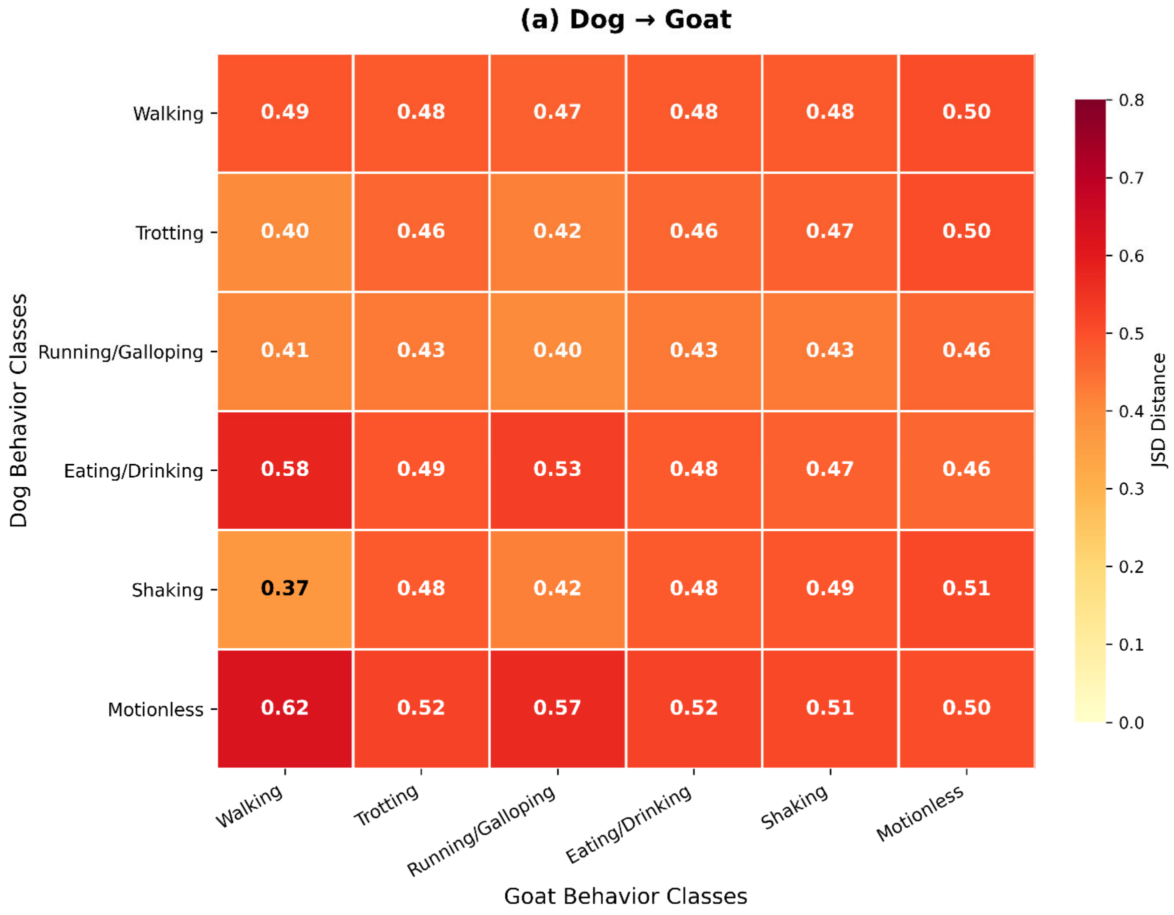

Figure S5. Unified ontology-class-level mean JSD distance matrix for the Dog → Horse transfer setting.

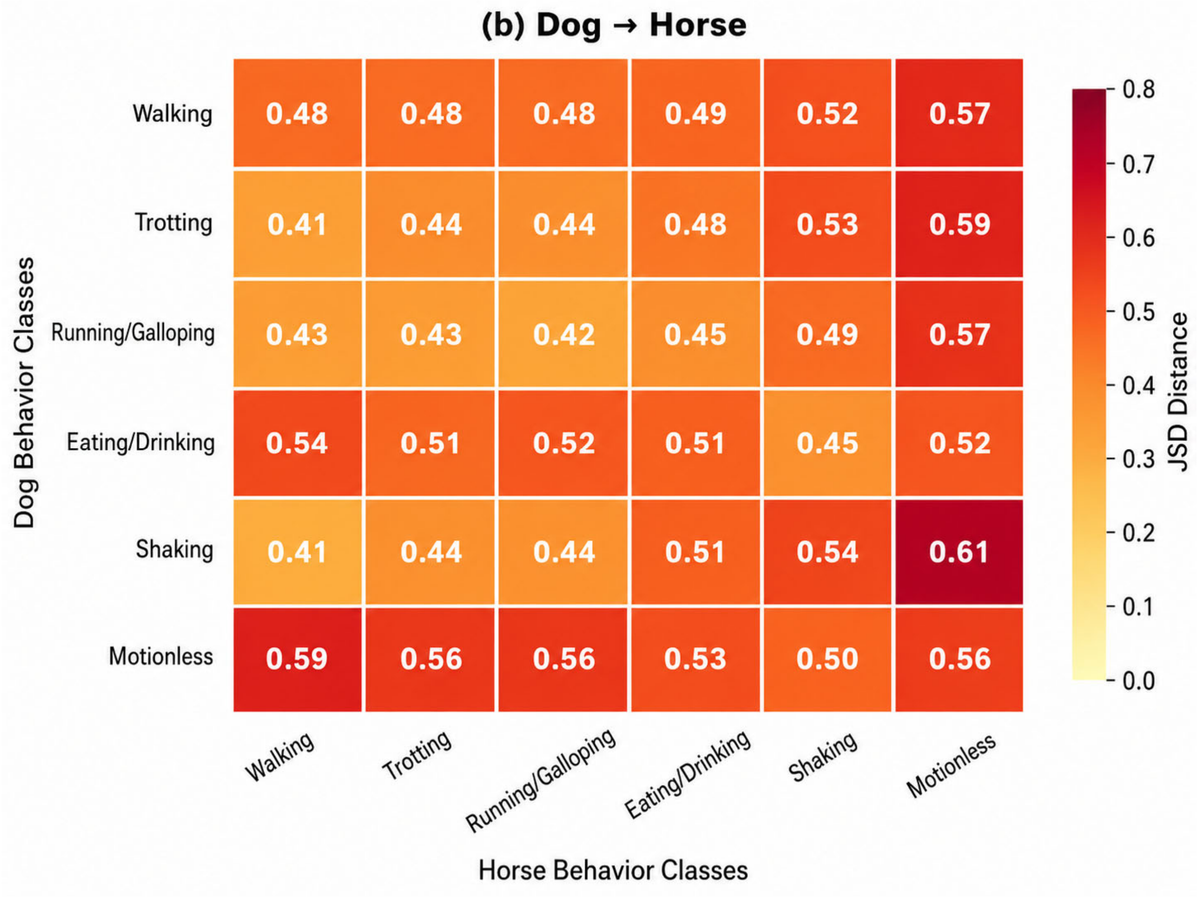

Figure S6. Unified ontology-class-level mean JSD distance matrix for the Goat → Horse transfer setting.

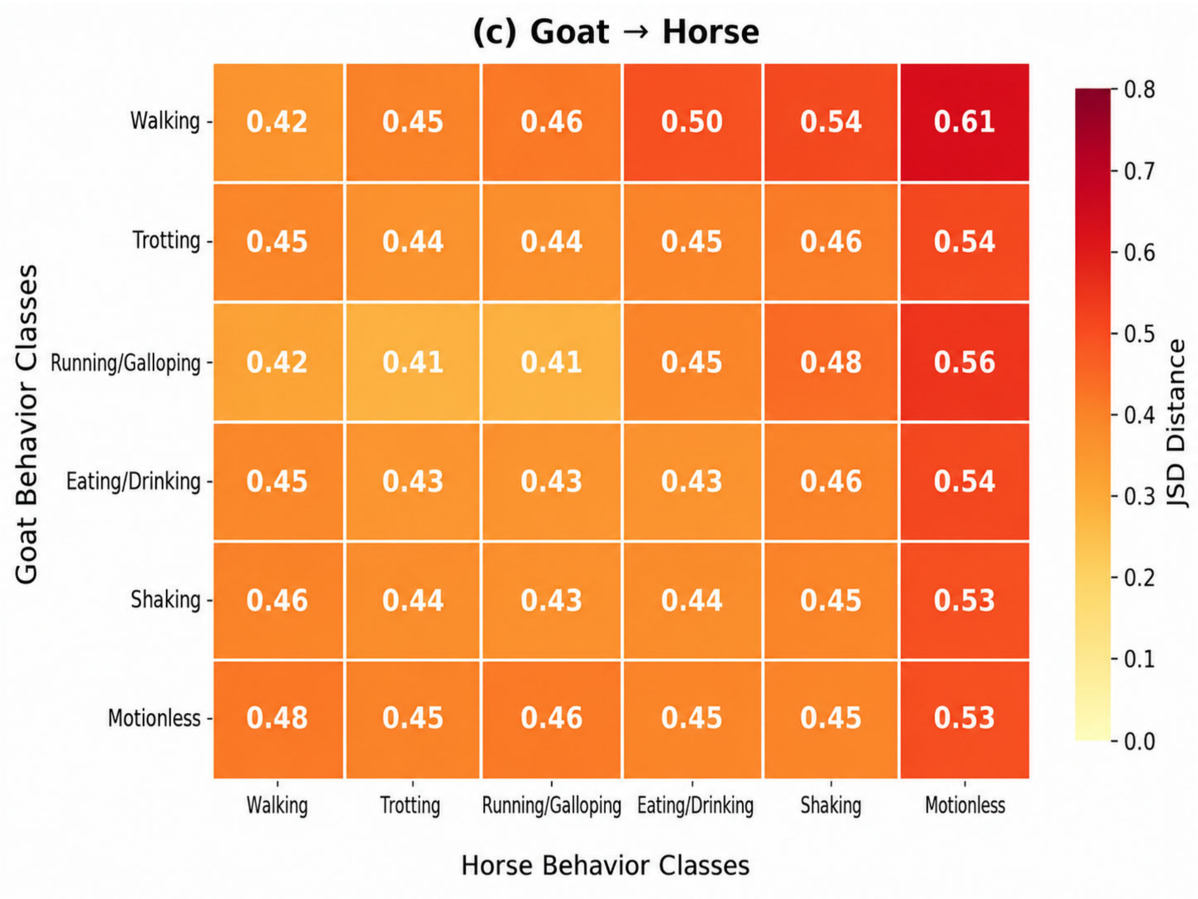

Table S1. Scenario-wise top five SHAP-ranked features across source–target transfer settings.

Source species	Target species	Architecture	Top five SHAP-ranked features
Dog	Goat	BiLSTM-Attention	Gz_zcr, Gy_zcr, Gx_zcr, Ax_zcr, Gx_dom_ratio
Dog	Goat	GRU-Attention	Ax_zcr, Gx_zcr, Gy_zcr, Gz_zcr, Ay_zcr
Dog	Goat	Transformer	Gz_zcr, Gx_dom_ratio, Gx_zcr, Gy_zcr, Gz_dom_ratio
Dog	Goat	CNN1D	Ay_zcr, Ax_zcr, Gy_zcr, Gx_zcr, Gz_zcr
Dog	Horse	BiLSTM-Attention	Gz_zcr, Ax_zcr, Gx_dom_ratio, Gx_zcr, Gy_zcr
Dog	Horse	GRU-Attention	Gz_zcr, Gz_dom_freq, Ax_zcr, Gx_dom_ratio, Az_band35
Dog	Horse	Transformer	Az_kurt, Az_band35, Gz_rms, Ay_rms, Az_dom_freq
Dog	Horse	CNN1D	Gz_zcr, Gy_zcr, Gx_zcr, Gy_dom_ratio, Ax_zcr
Goat	Dog	BiLSTM-Attention	Gz_zcr, Gx_zcr, Gy_zcr, Ax_zcr, Gx_dom_ratio
Goat	Dog	GRU-Attention	Gz_zcr, Gx_zcr, Gx_dom_ratio, svm_acc_mean, Gy_zcr
Goat	Dog	Transformer	Gz_zcr, Gx_dom_ratio, Gx_zcr, Gz_rms, svm_acc_mean
Goat	Dog	CNN1D	Ay_zcr, Ax_zcr, Gx_zcr, Gy_zcr, Gx_dom_ratio
Goat	Horse	BiLSTM-Attention	Gz_zcr, Gx_dom_ratio, Gy_zcr, Gx_zcr, Ax_zcr
Goat	Horse	GRU-Attention	Ax_zcr, Gz_zcr, Gx_zcr, Gx_dom_ratio, Ay_zcr
Goat	Horse	Transformer	Gz_zcr, Gx_dom_ratio, Gy_zcr, Gx_zcr, Ax_zcr
Goat	Horse	CNN1D	Ay_zcr, Gy_dom_ratio, Gz_dom_ratio, Gx_dom_ratio, Gx_zcr
Horse	Dog	BiLSTM-Attention	Gz_zcr, Gx_zcr, Gy_zcr, Gx_dom_ratio, Gz_dom_ratio
Horse	Dog	GRU-Attention	Gz_zcr, Gx_zcr, Gx_dom_ratio, Gy_zcr, Gy_dom_ratio
Horse	Dog	Transformer	Gz_zcr, Gx_zcr, Gx_dom_ratio, Gy_zcr, Gz_dom_ratio
Horse	Dog	CNN1D	Gy_zcr, Gz_zcr, Ax_zcr, Ay_zcr, Az_zcr
Horse	Goat	BiLSTM-Attention	Gz_zcr, Gy_zcr, Gx_zcr, Ax_zcr, Gx_dom_ratio
Horse	Goat	GRU-Attention	Gz_zcr, Gy_zcr, Gx_zcr, Gx_dom_ratio, Ax_zcr
Horse	Goat	Transformer	Gx_zcr, Gz_zcr, Gx_dom_ratio, Gy_zcr, Ay_zcr
Horse	Goat	CNN1D	Ay_zcr, Gx_zcr, Gz_zcr, Ax_zcr, Gy_zcr

Table S1 reports the five most influential features identified by SHAP for each source–target transfer scenario and model architecture. Across most scenarios, zero-crossing-rate features derived from gyroscope signals, particularly Gz_zcr, Gx_zcr, and Gy_zcr, consistently appear among the highest-ranked predictors. This pattern indicates that rotational motion dynamics are central to distinguishing cross-species behavioral patterns. In addition, dominant-frequency and dominant-ratio features, such as Gx_dom_ratio, Gz_dom_ratio, and Gz_dom_freq, contribute substantially in several transfer settings, suggesting that frequency-domain characteristics provide complementary information to time-domain movement irregularity. The repeated occurrence of accelerometer-based features, including Ax_zcr, Ay_zcr, Az_band35, and Az_kurt, further shows that linear acceleration patterns support model interpretability, especially in dog-to-horse and goat-to-horse transfer scenarios. Overall, the SHAP results demonstrate that the proposed models rely primarily on interpretable motion-related descriptors rather than arbitrary or species-specific artifacts.
